# Associations Between Pre-Quarantine Exercise and Persistent Symptoms After SARS-CoV-2 Infection

**DOI:** 10.3390/sports14070293

**Published:** 2026-07-09

**Authors:** Nikola Schmidt, Kira Engl, Barbara Grüne, Annelene Kossow, Johannes Nießen, Stefanie Wessely, Luis Haberstock, Susanne Rost, Christine Joisten

**Affiliations:** 1 Department for Physical Activity in Public Health, Institute of Movement and Neurosciences, German Sport University Cologne, 50933 Cologne, Germanyc.joisten@dshs-koeln.de (C.J.); 2 Department of Infection Control and Environmental Hygiene, Cologne Health Authority, 50667 Cologne, Germanybarbara.gruene@stadt-koeln.de (B.G.); annelene.kossow@stadt-koeln.de (A.K.); johannes.niessen@bioeg.de (J.N.); 3 Institute of Hygiene, University Hospital Muenster, 48149 Muenster, Germany; 4 Augsburg County Health Authority, 86150 Augsburg, Germany; luis.haberstock@lra-a.bayern.de (L.H.); susanne.rost@lra-a.bayern.de (S.R.)

**Keywords:** long-term symptoms, persistent symptoms, post-COVID-19 condition, long COVID, exercise, physical activity, predictors

## Abstract

Background: Long-term symptoms after SARS-CoV-2 infection such as fatigue, shortness of breath and cognitive impairment represent a major burden on society. Risk factors include female sex, smoking, comorbidities and socioeconomic deprivation. Physical activity (PA) has been suggested as a potential protective factor, although all population groups, including athletes, were affected. Therefore, this study aimed to investigate the influence of PA duration and intensity on the odds of the presence of long-term symptoms. Methods: This cross-sectional study included 5413 individuals following acute COVID-19 within the CoCo-Fakt online monitoring study. The type, duration and intensity of exercise in the four weeks before quarantine were recorded, and the odds of the presence of long-term symptoms beyond 12 weeks after infection were determined, adjusted for demographics, health status and acute COVID-19 outcomes. Results: Among participants, 561 (10.4%) reported long-term symptoms. Those with long-term symptoms reported a longer duration (*p* = 0.019, *d* = −0.61) of exercise in the four weeks before quarantine compared to those without long-term symptoms. Adjusted for demographics, health status and acute COVID-19 outcomes, higher exercise intensity (MET/day) was associated with 16.7% increased odds of long-term symptoms (Nagelkerke *R*^2^ = 18.0%). After the Bonferroni–Holm correction, this association did not remain significant. Conclusions: Current data suggests that PA has a protective effect on post-COVID-19 condition when performed at a moderate level. In our study, however, neither PA intensity nor duration emerged as a predictor of long-term symptoms. Future studies must clarify which intensities and types of exercise can help to maintain overall physical and mental health and to prevent or improve the long-term outcomes following SARS-CoV-2 infection, considering individual circumstances.

## 1. Introduction

Although the coronavirus disease 2019 (COVID-19) pandemic has now become endemic, patients are still affected by the long-term effects of severe acute respiratory syndrome coronavirus 2 (SARS-CoV-2) infection. A recent meta-analysis by Hou et al. found that the global prevalence of post-COVID-19 condition is 36% (33–40%) among individuals with a confirmed COVID-19 diagnosis [1]. However, its exact spread cannot yet be reliably determined due to its nonspecific manifestation, lack of biomarkers and limitations in current studies [2,3].

According to the World Health Organization (WHO)’s definition, post-COVID-19 condition presents with various physical, mental and cognitive symptoms that usually develop three months after SARS-CoV-2 infection and last for at least two months [3,4]. Symptoms can develop anew after initial recovery or persist after infection and cannot be explained by another diagnosis. Common symptoms include fatigue, shortness of breath, cognitive impairment and headache [5,6], which can lead to long-term absence from work, thereby imposing a social burden with high costs [7] and an increased individual burden due to a reduced quality of life [8], impaired cognitive and physical function and diminished participation in society [9].

Risk factors for post-COVID-19 condition include symptomatic acute COVID-19 courses, female sex, smoking, comorbidities and socioeconomic deprivation [10]. Limited research has explored vaccination [11], higher education [12,13] and living in urban areas [13] as possible protective factors for post-COVID-19 condition. Physical activity (PA) is considered a factor that not only contributes to prevention of SARS-CoV-2 infection [14] but that can also, when performed regularly prior to infection, lead to better outcomes in individuals with acute COVID-19, such as shorter recovery time, lower rates of hospitalization and severe disease course [15,16,17,18,19]. Furthermore, exercise training in rehabilitation programs seem to play a beneficial role in the recovery of respiratory symptoms [20]. Possible explanations for this observation include the link between physical inactivity and chronic diseases, which are associated with more severe disease progression, as well as inflammatory processes [21,22]. Concerning several manifestations of post-COVID-19 condition, a persistent increase in inflammatory cytokines, which could cause various systemic and organ-specific impacts, has been discussed [23]. Conversely, a healthy lifestyle including high PA levels, a healthy diet and non-smoking could reduce the risk of long-term symptoms due to anti-inflammatory mechanisms. Some studies have already shown an association between the risk of long-term symptoms and PA [24,25] or a healthy lifestyle [26], albeit with an exclusively female cohort, a lack of differentiation in intensity and a small sample size. However, one study involving a cohort of fit young adults found a prevalence of post-COVID-19 condition ranging from 1 to 4%, which, at that time, was comparable to the average prevalence of around 2% in individuals aged 18–24 years [27].

As part of the CoCo-Fakt (Cologne-Corona-Beratung und Unterstützung Für Index- und KontAKt-Personen während der Quarantäne-ZeiT [Cologne-Corona counseling and support for index and contacts during the quarantine period—author’s translation]) cross-sectional study, we investigated the association of PA duration and intensity and long-term symptoms after SARS-CoV-2 infection. We hypothesized that engaging in physical activity four weeks before quarantine would decrease the odds of the presence of long-term symptoms.

## 2. Materials and Methods

### 2.1. Study Design

The CoCo-Fakt study is a cross-sectional, mixed-methods online survey, conducted in three waves with individuals infected with SARS-CoV-2 (confirmed by quantitative real-time polymerase chain reaction or rapid testing from the second wave onwards) and their contacts in Cologne (first to third waves) and the county of Augsburg (third wave). Inclusion criteria were a minimum age of 16 years and a valid email address. Deceased individuals, individuals in medical or nursing facilities, and non-compliant individuals were excluded. All participants provided informed consent prior to participation.

Corresponding cases were reported to the health departments, where they were entered into a database [28], and isolated or quarantined for the applicable period in accordance with the German Infection Protection Act.

The survey waves included individuals who were in isolation or quarantine from the start of the COVID-19 pandemic until 9 December 2020 (first wave, not included here), between 1 January and 30 June 2021 (second wave), and between 1 July 2021 and 31 December 2021 (third wave). Individuals who met the inclusion criteria for the CoCo-Fakt study were extracted from the databases and received an invitation via email with a link to the online survey. They were sent a reminder two and four weeks after the invitation. The email addresses were not linked to the survey data at any time, ensuring that the survey was completely anonymized.

The survey was developed and modified based on the COVID-19 Snapshot Monitoring (COSMO) study [29] and conducted using the Unipark survey tool (Tivian XI GmbH, Munich, Germany) [30]. Individuals who were pregnant and their partners received a supplement to the questionnaire containing pregnancy-related questions. Individuals who stated that they had children were redirected to a children’s questionnaire after completing the survey. The study protocol of the first wave has been published previously [31]. For the second and third waves, the survey was modified according to arising knowledge during the pandemic (Supplementary File S1). Completing the questionnaire took approximately 30–45 min.

### 2.2. Study Sample

**Figure 1:**
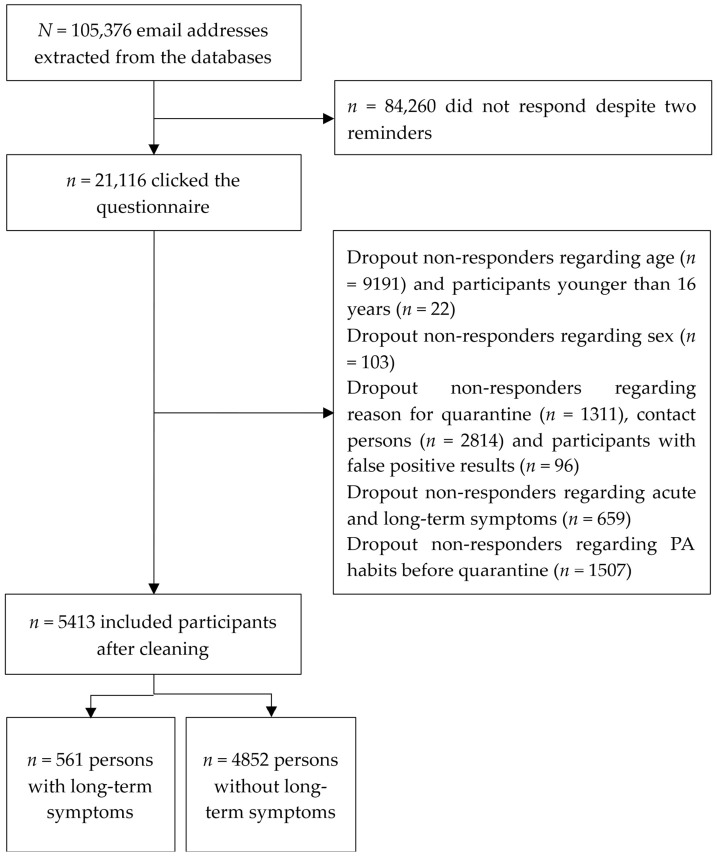
Study population flow chart.

Since no common definition existed for post-COVID-19 condition at the time of the first wave, only data from the second and third waves were evaluated in this analysis. A total of 105,376 individuals who were infected with SARS-CoV-2 or had been a contact person between 1 January and 31 December 2021 were contacted. Of these, 21,116 individuals clicked the questionnaire. This analysis included 5413 individuals who were aged at least 16 years, were in quarantine as infected individuals, and provided information on sex, acute and long-term symptoms after SARS-CoV-2 infection and PA habits before quarantine (Figure 1).

### 2.3. Survey

#### 2.3.1. Demographic Data

Participants’ age (in years), sex (female or male), socioeconomic status (SES) and migration background (no or yes) were recorded. SES was calculated from participants’ school and vocational education and classified into ‘low’, ‘medium’ or ‘high’ according to the first wave of the German Health Interview and Examination Survey for Adults [32]. Due to small numbers, the ‘low’ and ‘medium’ categories were combined. Migration background was classified based on the language predominantly spoken at home (German = no, all other languages = yes).

#### 2.3.2. Health Status

The participants were asked to report their height (cm) and weight (kg), which was used to calculate their body mass index (BMI) and classify them as underweight, normal weight, overweight or obese according to WHO guidelines [33]. We further recorded whether one or more of the following chronic diseases were present: pulmonary disease, cardiovascular disease, neurological disease, musculoskeletal disease, metabolic disease, chronic gastrointestinal disease, chronic kidney problems or kidney failure, mental illness, dermatological disease, cancer, allergies, autoimmune disease, or other. Based on the information provided for ‘other’, the participants were classified into further categories (gynecological disease, hematological or infectious disease, ophthalmological disease, otorhinolaryngologic disease, rheumatic disease, and vascular disease).

#### 2.3.3. COVID-19-Related Questions

We recorded the participants’ COVID-19 vaccination status (vaccinated once, twice or three times; infected and vaccinated once, twice or three times; not vaccinated yet, but would like to be vaccinated; does not want to be vaccinated) and then categorized them as “vaccinated” or “unvaccinated”. We also assessed all SARS-CoV-2 mutations known at the time of the survey (no mutation, Alpha [B.1.1.7], Beta [B.1.351], Gamma [P.1], Delta [B.1.617.2], multiple mutations, unknown, and other) and categorized them as ‘virus mutation present’ or ‘no virus mutation present’.

We asked the participants about the severity of their acute symptoms (‘Completely symptom-free’; ‘Mild symptoms, on how many days?’; ‘Significant symptoms, on how many days?’; ‘Severe feeling of illness, on how many days?’; ‘Illness-related anxiety’; ‘Long-term symptoms [post-COVID-19 condition/>4 weeks]’; and ‘Other’, modified according to [34]). When reporting symptoms, we determined the respective duration (in days) and intensity (‘did not occur’, ‘mild symptoms’, ‘moderate symptoms’, and ‘severe symptoms’) for nine symptoms frequently described in the literature (fever > 38 °C, cough, loss of appetite, fatigue, mild flu symptoms, muscular exhaustion, diarrhea, shortness of breath, and headache) [35] within the first two weeks after the positive test. Based on the type and severity, we classified the symptoms as follows and ranked them according to the most severe manifestation:

- Asymptomatic course (‘completely symptom-free’);
- Mild course: any intensity of loss of appetite or mild other symptoms;
- Moderate course: severe or moderate symptoms of cough, fatigue, muscular exhaustion, headache or diarrhea, or moderate symptoms of fever, flu or shortness of breath;
- Severe course: severe symptoms of fever, flu or shortness of breath.

If the participants reported long-term symptoms (indication of ‘post-COVID-19 condition/>4 weeks’ in the acute symptoms section), they were asked which of the most common post-COVID-19 condition symptoms (fatigue, cognitive impairment [‘brain fog’, loss of concentration or memory issues], loss of taste, loss of smell, respiratory symptoms [breathlessness, shortness of breath], psychological or psychiatric symptoms [depression, anxiety or post-traumatic stress disorder], sleep disturbance, muscular exhaustion, headaches, hair loss, and other) [36] were still present or had newly occurred at least four weeks after the positive test. For each of the listed symptoms, the course (‘no symptoms’, ‘mild symptoms’, ‘moderate symptoms’, or ‘severe symptoms’) was asked in relation to the period of occurrence (up to four weeks after illness, four to eight weeks after illness, more than eight weeks to less than three months, and more than three months). If the participants reported symptoms beyond 12 weeks after illness, they were assigned to the group ‘with long-term symptoms’. If they either reported not having post-COVID-19 condition in the acute symptom section or being asymptomatic beyond 12 weeks after illness, they were assigned to ‘without long-term symptoms’.

The long-term symptoms were then summarized for periods of four weeks to less than three months and more than three months, classified according to the type and intensity of the symptoms as follows, and assigned to the most severe category:

- Mild course: any intensity of loss of smell, loss of taste or hair loss, or mild other symptoms;
- Moderate course: severe or moderate symptoms of cognitive impairment, sleep disturbance, muscular exhaustion or headache, or moderate respiratory symptoms or fatigue;
- Severe course: severe symptoms of respiratory symptoms or fatigue.

#### 2.3.4. Physical Activity

We recorded participants’ daily life activities (e.g., gardening, occupational or home activities), exercise and relaxation habits four weeks before and during quarantine, as well as in the last two weeks before the survey (second wave only, modified according to [37]). In the current analysis, only PA habits before quarantine were evaluated; all other information, including daily life activities, was excluded. The participants were asked about the types of activity, frequencies (days/week) and number of minutes per day for both moderate and vigorous activities. A general metabolic equivalent of task (MET) value was derived for each sporting activity based on the Compendium of Physical Activities [38]. Where possible, differentiated MET values were assigned for moderate and vigorous activities. From this information, duration (minutes/week) and intensity (MET/week) were summed for moderate, vigorous and total PA (moderate and vigorous activities) and a mean score of minutes/day and MET/day calculated.

### 2.4. Data Analysis

We calculated the frequency (percentage), central tendency (mean) and variation (standard deviation [SD]) to describe the sample. Comparisons between participants with and without long-term symptoms as well as their demographic (age, sex, migration background, SES, BMI, chronic diseases, and smoking status) and COVID-related characteristics (COVID-19 vaccination status, SARS-CoV-2 mutations, and severity and duration of the acute COVID-19 course), respectively, were analyzed descriptively using Pearson’s *χ*^2^ tests and independent *t*-tests. Effect sizes for significant differences were calculated using Cramer’s *V* (Pearson’s *χ*^2^ test; small: 0.06–0.15; moderate: 0.16–0.26; large ≥ 0.26) or Cohen’s *d* (*t*-test; trivial: <0.2; small: 0.2–0.5; moderate: 0.5–0.8; large: ≥0.8).

To determine the effects of lifestyle on symptoms, we performed binary logistic regression analyses. The covariates included age, sex, migration background, SES, BMI, chronic diseases, COVID-19 vaccination status, SARS-CoV-2 mutation, severity of acute symptoms, duration of acute symptoms and smoking status.

All analyses were performed using IBM SPSS Statistics (version 31.0; IBM Deutschland GmbH, Ehningen, Germany). *p* < 0.05 was considered statistically significant. To address the issue of multiple testing, *p*-values of the two regression models were corrected using the Bonferroni–Holm method.

## 3. Results

### 3.1. Sample Characteristics

Among participants, the mean age was 43.5 (SD 13.8) years, and 60.6% were female (Table 1). Further, 1430 (26.5%) reported having at least one chronic disease (Table A1). Long-term symptoms were reported by 561 participants (10.4%) and occurred more often in females (*p* < 0.001, *V* = 0.05), individuals with a higher BMI (both absolute and categories *p* < 0.001, *d* = −0.16, *V* = 0.06), individuals with chronic diseases (*p* < 0.001, *V* = 0.12) and the presence of a SARS-CoV-2 mutation (*p* = 0.012, *V* = 0.05). The effect sizes of all significant results were trivial to moderate. Sex-stratified sample characteristics are presented in Appendix A (Table A2).

### 3.2. Acute and Post-COVID-19 Condition Symptoms

**Figure 2:**
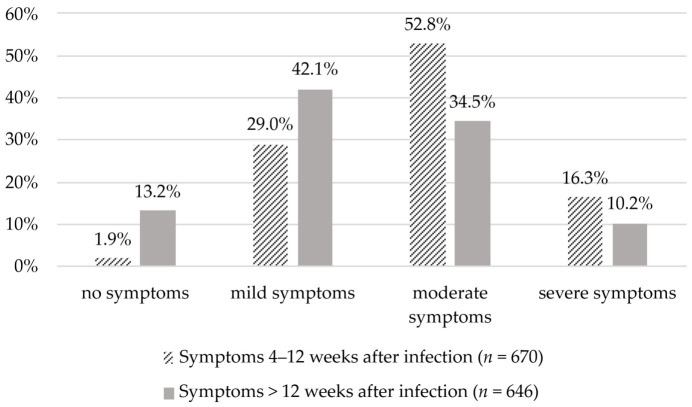
Symptom severity 4–12 weeks and >12 weeks after SARS-CoV-2 infection of participants with long-term symptoms.

Participants with long-term symptoms more often reported severe acute infection symptoms within the first two weeks (*p* < 0.001, *V* = 0.25) and a longer duration of acute symptoms (7.9 [SD 5.0 days] vs. 5.4 [SD 4.1 days], *p* < 0.001, *d* = −0.61, Table 2), both results with moderate effect sizes. Participants most frequently reported moderate symptoms within the first 4–12 weeks (*n* = 354, 52.8%, Figure 2), and mild symptoms beyond 12 weeks after infection (*n* = 272, 42.1%). The most commonly reported long-term symptoms beyond 12 weeks were fatigue (*n* = 374, 18.9%), cognitive impairment (*n* = 289, 14.6%) and respiratory symptoms (*n* = 242, 12.2%, Table A3). Sex- and age-stratified descriptive results on the presence of long-term symptoms are presented in Appendix A (Table A4).

### 3.3. Physical Activity

Among all participants, 3909 (72.2%) reported participating in PA in the four weeks before quarantine (Table 3). The mean PA duration was 43.7 (SD 38.5) minutes/day, and the mean intensity of PA was 1.3 (SD 1.3) MET/day. Participants with long-term symptoms reported a longer duration than those without long-term symptoms (49.5 [SD 43.6] vs. 43.0 [SD 37.9] MET/day, *p* = 0.019) with a trivial effect size (*d* = −0.17). Sex- and age-stratified descriptive results on PA participation, duration and intensity are presented in Appendix A (Table A4).

### 3.4. Regression Analyses

Binary logistic regression analyses revealed that female vs. male sex (OR: 0.668, 95% confidence interval [CI]: 0.463–0.965, *p* = 0.031), higher vs. lower BMI (OR: 1.037, 95% CI: 1.003–1.072, *p* = 0.034), having no migration background vs. having a migration background (OR: 0.375, 95% CI: 0.142–0.992, *p* = 0.048), chronic diseases (OR: 1.796, 95% CI: 1.242–2.598, *p* = 0.002), moderate versus asymptomatic/mild acute disease progression (OR: 2.689, 95% CI: 1.576–4.588, *p* < 0.001), severe versus asymptomatic/mild acute disease progression (OR: 4.010, 95% CI: 2.303–6.980, *p* < 0.001), longer versus shorter duration of acute symptoms (OR: 1.106, 95% CI: 1.058–1.156, *p* < 0.001), and higher versus lower PA intensities (OR: 1.167, 95% CI: 1.031–1.321, *p* = 0.015) increased the odds of the presence of long-term symptoms (Table A5). After the Bonferroni–Holm correction, this association did not remain statistically significant. The model explained 18.0% of the variance in long-term symptoms (Nagelkerke *R*^2^). Notably, PA duration showed no significant influence on the odds of the presence of long-term symptoms in the regression analysis (Table A6).

## 4. Discussion

### 4.1. Main Findings

This cohort study investigated the association between the presence of long-term symptoms and pre-quarantine exercise behavior. About 11% of individuals infected with SARS-CoV-2 reported symptoms beyond 12 weeks after infection. Fatigue and cognitive impairment were the most frequently reported symptoms, followed by respiratory symptoms. This observation aligns with the results of a previous meta-analysis on the most common general, neurological and cardiopulmonary symptoms [39]; however, the frequencies in our study were lower overall (fatigue: 19% vs. 29%; cognitive impairment: 15% vs. 29%; dyspnea: 12% vs. 21%).

Unexpectedly, participants with long-term symptoms reported a longer PA duration in the four weeks before quarantine than those without long-term symptoms, albeit with a trivial effect size. Adjusted for demographics, health status and COVID-19–specific parameters, the main predictors of the presence of long-term symptoms were chronic diseases, acute COVID-19 symptom severity and longer duration of acute symptoms.

In the context of long-term symptoms, the evidence on the role of PA is currently sparse and inconsistent. Feter et al. showed that PA reduced the odds of fatigue, neurological complications, coughing and headaches; however, PA did not affect the overall incidence of post-COVID-19 condition [24]. Another study examined six lifestyle factors (BMI, smoking, diet, alcohol consumption, PA and sleep) in relation to the risk of post-COVID-19 condition in 2000 women [26], showing a 50% reduced risk of post-COVID-19 condition in those with five or six healthy lifestyle factors, adjusted for sociodemographic factors and pre-existing conditions. Looking at PA separately, inactive individuals (0 to 30 min of moderate or vigorous PA per week) were at a 20% increased risk of post-COVID-19 condition compared to those who were active for more than 210 min per week, adjusted for age, race and ethnicity, and SES. However, in the further model that also adjusted for healthcare worker status and chronic disease history, no effects of PA were found.

Athletes may experience greater limitations than inactive individuals due to their enhanced body awareness and greater physiological demands. Vollrath et al. showed that 70% of athletes still exhibited related symptoms 4.1 (SD 3.8) months after acute SARS-CoV-2 infection and although this proportion dropped to 62% three months later [40], the number remained considerably higher than the 36% reported by the general population in other studies [1]. At both examination points, fatigue and performance decrease were most frequently reported among symptomatic athletes. However, even asymptomatic athletes showed a deterioration in performance metrics due to the acute infection. Another study reported that although only 10% of Olympic athletes remained symptomatic one month after SARS-CoV-2 infection, the infection appeared to have a more pronounced impact on return to full sport participation as more than a quarter of athletes were affected [41]. Since our study examined self-reported symptoms without a medical diagnosis, a possible explanation might be that a sustained decline in performance is interpreted as persistent symptom, such as fatigue. Although most physically active individuals experience asymptomatic or mild courses of the disease, those who engage in high-intensity exercise training place greater demands on their physiological functioning in order to regain their pre-infection level of performance.

Additionally, post-COVID-19 condition is characterized by persistent inflammation and immunological dysregulation, such as elevated levels of conventional inflammatory mediators, sometimes lasting for months [42], which may contribute to the clinical picture, including fatigue. Evidence suggests persistent activation of the innate immune system, including increased monocyte–platelet aggregates and imbalances in the complement system, as well as associated thromboinflammatory processes and endothelial dysregulation [43]. Concurrently, dysregulation of adaptive immunity has been described, characterized by the expression of exhaustion markers by cluster of differentiation 8 (CD8)^+^ and 4 (CD4)^+^ T cells [44] and a reduction in naïve T cells [45], indicating persistent immunological stimulation.

Regular moderate PA has beneficial effects on the immune system through activation of anti-inflammatory signaling pathways like the release of myokines such as interleukin (IL)6, with downstream induction of IL10 and inhibition of tumor necrosis factor [46]. Contrary, high-intensity exercise can lead to transient immune depression resulting in a higher infection risk during these periods. However, since no biomedical parameters were measured in our study, the interpretation remains speculative.

Next to performance measures and immunological parameters, engaging in PA is known to be beneficial for overall physical and mental health. The measures taken to mitigate the viral spread, as well as the disease itself, led to a reduction in PA across all age groups [47] as well as a decrease in mental health outcomes [48]. One study found that particularly physically active individuals were negatively affected by home confinement and the accompanying reduction in PA compared to physically inactive individuals, as they experienced a significant decline in sleep quality and general well-being [49]. Nevertheless, returning to pre-pandemic PA levels is crucial for all individuals, as especially individuals with chronic diseases are at increased risk for long-term symptoms following COVID-19 [10]. However, long-term reductions in PA were observed specifically among people with chronic conditions [50] and those who had severe courses of COVID-19 [51]. Therefore, future studies are crucial to investigate negative long-term effects of reduced PA on physical and mental health and to gain a better understanding of the effects of high-intensity PA on long-term symptoms associated with COVID-19. These studies should consider differentiated approaches to the intensity of PA, the length of recovery phases, individual adaptation to symptom dynamics and the monitoring of mental health outcomes.

### 4.2. Strengths and Limitations

This study’s strengths include broad recruitment from both urban and rural areas as well as a comprehensive survey capturing diverse influencing factors.

However, this study also had limitations that must be considered. Firstly, the questionnaire took around 30–45 min to complete, which may have led to incomplete responses or fatigue. Secondly, the questionnaire was predominantly completed by individuals with high SES, which is associated with positive lifestyle factors; therefore, a potential selection bias toward more health-conscious respondents cannot be ruled out. Thirdly, some surveys were completed long after infection, which may have led to misreporting. Fourthly, the lack of objective activity monitoring may have led to recall bias, self-report inflation and socially desirable responses. Seventy-four percent of participants reported meeting the WHO’s PA recommendations of at least 150 min per week (Table A2). That proportion is higher than national data from 2019/2020, which was 45% of women and 51% of men [52], suggesting overestimation. Further, MET values of the sporting activities were derived using the Compendium of Physical Activities meaning that the MET values for some activities may not be sufficiently differentiated. Fifthly, information on long-term symptoms was based on self-reported information rather than medical diagnosis. According to the WHO definition, symptoms of post-COVID-19 condition also last at least for two months and cannot be explained by another diagnosis which we both did not assess. Therefore, comparisons with studies on post-COVID-19 condition are limited. Finally, the questionnaire only covered the most common of the over 200 symptoms of post-COVID-19 condition [36].

## 5. Conclusions

PA appears to play a dual role in post-COVID-19 condition. While immunological and epidemiological data suggest that moderate PA has protective effects, studies on athletes show prolonged recovery phases in terms of their sporting activities. In our study, neither PA intensity nor duration showed an association with the presence or absence of long-term symptoms. Future studies must clarify which intensities and types of exercise can help to maintain overall physical and mental health and to prevent or improve the long-term outcomes after SARS-CoV-2 infection, considering individual circumstances.

## Figures and Tables

**Table 1:** Sample characteristics of participants with versus those without long-term symptoms.

	Total	Participants Without Long-Term Symptoms	Participants with Long-Term Symptoms	*p*	Effect Size
**Sample, *n* (%)**	5413	4852 (89.6%)	561 (10.4%)	-	-
**Age (years), mean (SD), range**	43.5 (13.8) 16–99	43.4 (13.9) 16–99	43.8 (13.4) 17–81	0.558 ^a^	-
**Sex, *n* (%)**					
Female	3279 (60.6%)	2896 (59.7%)	383 (68.3%)	<0.001 ^b^	0.05 ^c^
Male	2134 (39.4%)	1956 (40.3%)	178 (31.7%)		
**Height (cm), mean (SD), range**	172.5 (9.2) 142.0–206.0	172.6 (9.3) 142.0–206.0	171.2 (8.8) 150.0–200.0	<0.001 ^a^	0.15 ^d^
**Weight (kg), mean (SD), range**	78.3 (18.5) 39.0–190.0	78.1 (18.4) 39.0–190.0	79.5 (19.4) 46.0–167.0	0.104 ^a^	-
**BMI (kg/m^2^), mean (SD), range**	26.2 (5.3) 14.7–61.3	26.1 (5.3) 14.7–61.3	27.0 (5.8) 16.5–55.8	<0.001 ^a^	−0.16 ^d^
**BMI classification, *n* (%)**					
Underweight	91 (1.7%)	79 (1.7%)	12 (2.2%)	<0.001 ^b^	0.06 ^c^
Normal weight	2480 (46.6%)	2254 (47.2%)	226 (40.9%)		
Overweight	1746 (32.8%)	1569 (32.9%)	177 (32.1%)		
Obesity	1009 (18.9%)	872 (18.3%)	137 (24.8%)		
**Migration background (yes), *n* (%)**	340 (6.4%)	303 (6.4%)	37 (6.7%)	0.773 ^b^	-
**SES, *n* (%)**					
Low/medium	1869 (37.7%)	1676 (37.7%)	193 (37.4%)	0.890 ^b^	-
High	3091 (62.3%)	2768 (62.3%)	323 (62.6%)		
**Chronic disease (yes), *n* (%)**	1430 (26.5%)	1198 (24.7%)	232 (41,5%)	<0.001 ^b^	0.12 ^c^
**Smoker (yes), *n* (%)**	737 (16.0%)	650 (15.7%)	87 (18.3%)	0.155 ^b^	-
**COVID-19 vaccination (yes), *n* (%)**	3922 (73.0%)	3540 (73.4%)	382 (69.1%)	0.030 ^b^	0.03 ^c^
**SARS-CoV-2 mutation (yes), *n* (%)**	2087 (71.8%)	1778 (71.0%)	309 (77.1%)	0.012 ^b^	0.05 ^c^

Note: BMI = body mass index; COVID-19 = coronavirus disease 2019; SARS-CoV-2 = severe acute respiratory syndrome coronavirus 2; SES = socioeconomic status. ^a^ unpaired *t*-test. ^b^ *χ*^2^ test. ^c^ Cramer’s *V*. ^d^ Cohen’s *d*.

**Table 2:** Acute COVID-19 symptoms of recovered participants versus those with post-COVID-19 condition.

	Total	Participants Without Long-Term Symptoms	Participants with Long-Term Symptoms	*p*	Effect Size
**Presentation of acute COVID-19 symptoms, *n* (%)**					
Asymptomatic	451 (8.5%)	450 (9.4%) *	1 (0.2%)	<0.001 ^a^	0.25 ^b^
Mild	1613 (30.5%)	1561 (32.6%) *	52 (10.6%)		
Moderate	1783 (33.8%)	1642 (34.3%) *	141 (28.8%)		
Severe	1434 (27.2%)	1138 (23.8%) *	296 (60.4%)		
**Duration of acute COVID-19 symptoms (days), mean (SD), range**	5.6 (4.2) 0–14	5.4 (4.1) 0–14	7.9 (5.0) 0–14	<0.001 ^c^	−0.61 ^d^

Note: COVID-19 = coronavirus disease 2019. ^a^ *χ*^2^ test. ^b^ Cramer’s *V*. ^c^ unpaired *t*-test ^d^ Cohen’s *d*. * rounding error.

**Table 3:** PA participation, duration (min/day) and intensity (MET/day) in the four weeks before quarantine.

	Total	Participants Without Long-Term Symptoms	Participants with Long-Term Symptoms	*p*	Effect Size
**PA participation four weeks before quarantine, *n* (%)**					
Yes	3909 (72.2%)	3512 (72.4%)	397 (70.8%)	0.419 ^a^	–
No	1504 (27.8%)	1340 (27.6%)	164 (29.2%)		
**PA duration (minutes/day), mean (SD), range**	43.7 (38.5) 0.0–372.9	43.0 (37.9) 0.0–372.9	49.5 (43.6) 0.0–282.9	0.019 ^b^	−0.17 ^c^
**PA intensity (MET/day), mean (SD), range**	1.3 (1.3) 0.0–8.4	1.3 (1.3) 0.0–8.4	1.4 (1.4) 0.0–7.5	0.054 ^b^	–

Note: COVID-19 = coronavirus disease 2019; MET = metabolic equivalent of task; PA = physical activity. ^a^ *χ*^2^ test. ^b^ unpaired *t*-test. ^c^ Cohen’s *d*.

## Data Availability

The datasets used and/or analyzed during the current study are available from the corresponding author upon reasonable request. Requests for data sharing from appropriate researchers and entities will be considered on a case-by-case basis.

## Supplementary Materials

The following supporting information can be downloaded at: https://www.mdpi.com/article/10.3390/sports14070293/s1, Supplementary File S1: Survey of the second and third wave of the CoCo-Fakt study.

## Abbreviations

The following abbreviations are used in this manuscript:

BMI	Body mass index
CI	Confidence interval
CD	Cluster of differentiation
CoCo-Fakt	Cologne-Corona-Beratung und Unterstützung Für Index- und KontAKt-Personen während der Quarantäne-ZeiT (Cologne-Corona counseling and support for index and contacts during the quarantine period—author’s translation)
COVID-19	Coronavirus disease 2019
IL	Interleukin
LL	Lower limit
MET	Metabolic equivalent of task
OR	Odds ratio
PA	Physical activity
SARS-CoV-2	Severe acute respiratory syndrome coronavirus 2
SD	Standard deviation
SE	Standard error
SES	Socioeconomic status
UL	Upper limit
WHO	World Health Organization

## Appendix A

**Table A1:** The frequencies of chronic diseases in the study cohort.

Disease Type	Total	Participants Without Long-Term Symptoms	Participants with Long-Term Symptoms
Dermatological disease/allergies	564 (19.8%) *	472 (20.3%) *	92 (17.5%) *
Cardiovascular disease	510 (17.9%) *	424 (18.2%) *	86 (16.4%) *
Metabolic disease	480 (16.8%) *	408 (17.5%) *	72 (13.7%) *
Pulmonary disease	331 (11.6%) *	261 (11.2%) *	70 (13.3%) *
Musculoskeletal disorder	231 (8.1%) *	180 (7.7%) *	51 (9.7%) *
Mental illness	155 (5.4%) *	121 (5.2%) *	34 (6.5%) *
Neurological disorders	141 (4.9%) *	105 (4.5%) *	36 (6.9%) *
Autoimmune disease	136 (4.8%) *	110 (4.7%) *	26 (5.0%) *
Chronic gastrointestinal disease	133 (4.7%) *	100 (4.3%) *	33 (6.3%) *
Cancer	57 (2.0%) *	50 (2.2%) *	7 (1.3%) *
Rheumatic disease	27 (0.9%) *	23 (1.0%) *	4 (0.8%) *
Chronic kidney problems/kidney failure	22 (0.8%) *	19 (0.8%) *	3 (0.6%) *
Hematological/infectious disease	21 (0.7%) *	18 (0.8%) *	3 (0.6%) *
Gynecological disease	15 (0.5%) *	11 (0.5%) *	4 (0.8%) *
Vascular disease	14 (0.5%) *	12 (0.5%) *	2 (0.4%) *
Otorhinolaryngologic disease	7 (0.2%) *	6 (0.3%) *	1 (0.2%) *
Ophthalmological disease	6 (0.2%) *	5 (0.2%) *	1 (0.2%) *

Note: COVID-19 = coronavirus disease 2019. * rounding error.

**Table A2:** Sex-stratified sample characteristics.

	Total	Females	Males	*p*	Effect Size
**Sample, *n* (%)**	5413	3279 (60.6%)	2134 (39.4%)	–	–
**Age (years), mean (SD), range**	43.5 (13.8) 16–99	41.8 (13.4) 16–99	46.0 (14.2) 16–88	<0.001 ^a^	−0.30 ^b^
**Height (cm), mean (SD), range**	172.5 (9.2) 142.0–206.0	167.3 (6.4) 142.0–197.0	180.4 (7.0) 156.0–206.0	<0.001 ^a^	−1.97 ^b^
**Weight (kg), mean (SD), range**	78.3 (18.5) 39.0–190.0	71.3 (16.1) 39.0–175.0	88.9 (16.8) 43.0–190.0	<0.001 ^a^	−1.07 ^b^
**BMI (kg/m^2^), mean (SD), range**	26.2 (5.3) 14.7–61.3	25.5 (5.6) 15.6–61.3	27.3 (4.7) 14.7–51.5	<0.001 ^a^	−0.34 ^b^
**BMI classification, *n* (%)**					
Underweight	91 (1.7%)	84 (2.6%)	7 (0.3%)	<0.001 ^c^	0.24 ^d^
Normal weight	2480 (46.6%)	1763 (54.9%)	717 (33.9%)		
Overweight	1746 (32.8%)	812 (25.3%)	934 (44.2%)		
Obesity	1009 (18.9%)	552 (17.2%)	457 (21.6%)		
**Migration background (yes), *n* (%)**	340 (6.4%)	181 (5.6%)	159 (7.6%)	0.005 ^c^	0.04 ^d^
**SES, *n* (%)**					
Low/medium	1869 (37.7%)	1170 (38.9%)	699 (35.8%)	0.025 ^c^	0.03 ^d^
High	3091 (62.3%)	1836 (61.1%)	1255 (64.2%)		
**Chronic disease (yes), *n* (%)**	1430 (26.5%)	908 (27.8%)	522 (24.5%)	0.008 ^c^	0.04 ^d^
**Smoker (yes), *n* (%)**	737 (16.0%)	428 (15.4%)	309 (16.9%)	0.172 ^c^	–
**COVID-19 vaccination (yes), *n* (%)**	3922 (73.0%)	2377 (73.1%)	1550 (72.7%)	0.741 ^c^	–
**SARS-CoV-2 mutation (yes), *n* (%)**	2087 (71.8%)	1250 (71.5%)	837 (72.3%)	0.652 ^c^	–
**PA duration, *n* (%)**				<0.001 ^b^	0.06 ^c^
<150 min/week	824 (25.9%)	536 (28.1%)	288 (22.7%)		
≥150 min/week	2352 (74.1%)	1374 (71.9%)	978 (77.3%)		

Note: BMI = body mass index; COVID-19 = coronavirus disease 2019; SARS-CoV-2 = severe acute respiratory syndrome coronavirus 2; SES = socioeconomic status. ^a^ unpaired *t*-test. ^b^ *χ*^2^ test. ^c^ Cramer’s *V*. ^d^ Cohen’s *d*.

**Table A3:** The frequencies of long-term symptoms in the study cohort.

Long-Term Symptom	*n* (%)
Fatigue	374 (18.9%)
Cognitive impairment (‘brain fog’, loss of concentration or memory issues)	289 (14.6%)
Respiratory symptoms (breathlessness, shortness of breath)	242 (12.2%)
Muscle pain	189 (9.6%)
Sleep disturbance	181 (9.1%)
Headache	136 (6.9%)
Loss of smell	123 (6.2%)
Psychological/psychiatric symptoms (depression, anxiety or post-traumatic stress disorder)	123 (6.2%)
Hair loss	116 (5.9%)
Loss of taste	107 (5.4%)
Other	99 (5.0%)

Note: COVID-19 = coronavirus disease 2019.

**Table A4:** Sex- and age-stratified descriptive results on the presence of long-term symptoms, PA participation, PA duration (minutes/day) and PA intensity (MET/day).

Sex	Age Group	Long-Term Symptoms (Yes)	PA Participation (Yes)	PA Duration (Minutes/Day)		PA Intensity (MET/Day)	
		*n* (%)	*n* (%)	*n*	Mean (SD), Range	*n*	Mean (SD), Range
Female	16–29	83 (12.6%)	435 (66.1%)	322	46.1 (35.9) 0–244.3	410	1.7 (1.4) 0–7.3
	30–44	136 (10.6%)	897 (70.1%)	606	38.6 (38.0) 0–372.9	854	1.2 (1.3) 0–8.4
	45–59	124 (12.2%)	755 (74.3%)	535	40.5 (33.0) 0–231.4	740	1.2 (1.3) 0–8.2
	≥60	40 (12.3%)	245 (75.2%)	148	46.5 (49.3) 5.7–278.6	242	0.9 (1.1) 0–5.8
Male	16–29	17 (5.6%)	225 (74.0%)	163	60.2 (50.1) 0–368.6	209	1.7 (1.5) 0–7.2
	30–44	59 (8.7%)	497 (72.9%)	349	45.7 (37.1) 0–250	470	1.5 (1.4) 0–7.3
	45–59	80 (10.4%)	573 (74.8%)	384	42.5 (37.8) 0–368.6	560	1.2 (1.2) 0–7.5
	≥60	22 (5.8%)	282 (73.8%)	174	46.5 (37.8) 0–214.3	277	1.0 (1.1) 0–4.2

Note: MET = metabolic equivalent of task; PA = physical activity.

**Table A5:** Influence of the intensity of moderate and vigorous PA (MET/day) on the odds of the presence of long-term symptoms, adjusted for age, sex, migration background, SES, BMI, chronic diseases, COVID-19 vaccination, SARS-CoV-2 mutation, acute symptoms, symptom duration and smoking status, as determined by binary logistic regression.

	Regression Coefficient B	SE	*p*	OR	95% CI	
					LL	UL
**Age (years)**	−0.005	0.007	0.452	0.995	0.981	1.008
**Sex**						
Female	ref.					
Male	−0.403	0.187	0.031	0.668	0.463	0.965
**BMI (kg/m^2^)**	0.036	0.017	0.034	1.037	1.003	1.072
**Migration background (yes)**	−0.981	0.497	0.048	0.375	0.142	0.992
**SES**						
Low/medium	ref.					
High	0.145	0.209	0.487	1.157	0.767	1.744
**Chronic disease (yes)**	0.586	0.188	**0.002 ***	1.796	1.242	2.598
**Smoker (yes)**	−0.136	0.267	0.609	0.873	0.517	1.472
**COVID-19 vaccination (yes)**	−0.105	0.181	0.561	0.900	0.631	1.284
**SARS-CoV-2 mutation (yes)**	0.094	0.208	0.653	1.098	0.731	1.651
**Acute symptoms**						
Asymptomatic/mild	ref.					
Moderate	0.989	0.273	**<0.001 ***	2.689	1.576	4.588
Severe	1.389	0.283	**<0.001 ***	4.010	2.303	6.980
**Symptom duration (days)**	0.101	0.023	**<0.001 ***	1.106	1.058	1.156
**Total intensity of moderate and vigorous PA (MET/day)**	0.154	0.063	0.015	1.167	1.031	1.321
**Presence of long-term symptoms (yes)**	ref.					

Note: *n* = 1416; Nagelkerke *R*^2^ = 18.0%; BMI = body mass index; CI = confidence interval; COVID-19 = coronavirus disease 2019; LL = lower limit; MET = metabolic equivalent of task; ref. = reference; SARS-CoV-2 = severe acute respiratory syndrome coronavirus disease 2; SES = socioeconomic status; UL = upper limit; * *p* < 0.05 after the Bonferroni–Holm correction.

**Table A6:** The influence of duration of moderate and vigorous PA (minutes/day) on the odds of the presence of long-term symptoms, adjusted for age, sex, migration background, SES, BMI, chronic diseases, vaccination, SARS-CoV-2 mutation, acute symptoms, symptom duration and smoking status, as determined by binary logistic regression analysis.

	Regression Coefficient B	SE	*p*	OR	95% CI	
					LL	UL
**Age (years)**	−0.012	0.008	0.146	0.999	0.973	1.004
**Sex**						
Female	ref.					
Male	−0.360	0.211	0.088	0.698	0.461	1.055
**BMI (kg/m^2^)**	0.040	0.020	0.047	1.040	1.000	1.082
**Migration background (yes)**	−1.612	0.756	0.033	0.200	0.045	0.878
**SES**						
Low/medium	ref.					
High	0.097	0.242	0.689	1.102	0.685	1.771
**Chronic disease (yes)**	0.654	0.212	**0.002 ***	1.924	1.270	2.914
**Smoker (yes)**	0.032	0.326	0.921	1.033	0.545	1.958
**COVID-19 vaccination (yes)**	−0.129	0.207	0.532	0.879	0.586	1.318
**SARS-CoV-2 mutation (yes)**	−0.095	0.228	0.676	0.909	0.582	1.421
**Acute symptoms**						
Asymptomatic/mild	ref.					
Moderate	1.094	0.328	**<0.001 ***	2.985	1.570	5.674
Severe	1.529	0.340	**<0.001 ***	4.612	2.368	8.983
**Symptom duration (days)**	0.104	0.026	**<0.001 ***	1.110	1.055	1.167
**Total duration of moderate and vigorous PA (minutes/day)**	0.003	0.002	0.255	1.003	0.998	1.007
**Presence of long-term symptoms (yes)**	ref.					

Note: *n* = 1082; Nagelkerke *R*^2^ = 20.0%; BMI = body mass index; CI = confidence interval; COVID-19 = coronavirus disease 2019; LL = lower limit; ref. = reference; SARS-CoV-2 = severe acute respiratory syndrome coronavirus disease 2; SES = socioeconomic status; UL = upper limit; * *p* < 0.05 after the Bonferroni–Holm correction.

## References

[1] Hou Y., Gu T., Ni Z., Shi X., Ranney M.L., Mukherjee B. (2025). Global Prevalence of Long COVID, its Subtypes and Risk factors: An Updated Systematic Review and Meta-Analysis. Open Forum Infect. Dis..

[2] Tsilingiris D., Vallianou N.G., Karampela I., Christodoulatos G.S., Papavasileiou G., Petropoulou D., Magkos F., Dalamaga M. (2023). Laboratory Findings and Biomarkers in Long COVID: What Do We Know So Far? Insights into Epidemiology, Pathogenesis, Therapeutic Perspectives and Challenges. Int. J. Mol. Sci..

[3] Robert Koch-Institut (2023). RKI—Coronavirus SARS-CoV-2—Long COVID (Stand: 22.8.2023). https://www.rki.de/SharedDocs/FAQ/NCOV2019/FAQ_Liste_Gesundheitliche_Langzeitfolgen.html?nn=13490888.

[4] World Health Organization (2022). Post COVID-19 Condition (Long COVID). https://www.who.int/europe/news-room/fact-sheets/item/post-covid-19-condition.

[5] Bahmer T., Borzikowsky C., Lieb W., Schäfer A., Krist L., Fricke J., Scheibenbogen C., Rabe K.F., Maetzler W., Maetzler C. (2022). Severity, predictors and clinical correlates of Post-COVID syndrome (PCS) in Germany: A prospective, multi-centre, population-based cohort study. eClinicalMedicine.

[6] Leitner M., Pötz G., Berger M., Fellner M., Spat S., Koini M. (2024). Characteristics and burden of acute COVID-19 and long-COVID: Demographic, physical, mental health, and economic perspectives. PLoS ONE.

[7] Wissenschaftliches Institut der AOK (2024). Post-Covid und Long-Covid: Sinkende Zahl von Krankschreibungen, Aber Weiterhin Lange Berufliche Fehlzeiten der Betroffenen. https://www.wido.de/fileadmin/Dateien/Dokumente/News/Pressemitteilungen/2024/wido_bgf_pm_post-covid_und_long-covid_0224.pdf.

[8] Malik P., Patel K., Pinto C., Jaiswal R., Tirupathi R., Pillai S., Patel U. (2022). Post-acute COVID-19 syndrome (PCS) and health-related quality of life (HRQoL)-A systematic review and meta-analysis. J. Med. Virol..

[9] Tabacof L., Tosto-Mancuso J., Wood J., Cortes M., Kontorovich A., McCarthy D., Rizk D., Rozanski G., Breyman E., Nasr L. (2022). Post-acute COVID-19 Syndrome Negatively Impacts Physical Function, Cognitive Function, Health-Related Quality of Life, and Participation. Am. J. Phys. Med. Rehabil..

[10] Chuang H.-J., Lin C.-W., Hsiao M.-Y., Wang T.-G., Liang H.-W. (2024). Long COVID and rehabilitation. J. Formos. Med. Assoc..

[11] Wong M.C.-S., Huang J., Wong Y.-Y., Wong G.L.-H., Yip T.C.-F., Chan R.N.-Y., Chau S.W.-H., Ng S.-C., Wing Y.-K., Chan F.K.-L. (2023). Epidemiology, Symptomatology, and Risk Factors for Long COVID Symptoms: Population-Based, Multicenter Study. JMIR Public Health Surveill..

[12] Eduvirgem J., Bressan J., Hermsdorff H.H.M., Montenegro L.C., Brandão M.L., Neves A.A.T., da Silva L.S.A., Gerake-Dias T.A., Pimenta A.M. (2024). Risk and protective factors for Long COVID in Brazilian adults (CUME Study). Front. Med..

[13] Perlis R.H., Santillana M., Ognyanova K., Safarpour A., Lunz Trujillo K., Simonson M.D., Green J., Quintana A., Druckman J., Baum M.A. (2022). Prevalence and Correlates of Long COVID Symptoms Among US Adults. JAMA Netw. Open.

[14] Yang J., Li X., He T., Ju F., Qiu Y., Tian Z. (2022). Impact of Physical Activity on COVID-19. Int. J. Environ. Res. Public Health.

[15] Zhou A., Xia Y., Pi P., Wang Z., Huang H., Wang Y. (2024). Relationship between infection, physical and mental health and exercise habits of some Chinese residents after recovery from COVID-19. Sports Med. Health Sci..

[16] Sallis R., Young D.R., Tartof S.Y., Sallis J.F., Sall J., Li Q., Smith G.N., Cohen D.A. (2021). Physical inactivity is associated with a higher risk for severe COVID-19 outcomes: A study in 48 440 adult patients. Br. J. Sports Med..

[17] Ezzatvar Y., Ramírez-Vélez R., Izquierdo M., Garcia-Hermoso A. (2022). Physical activity and risk of infection, severity and mortality of COVID-19: A systematic review and non-linear dose-response meta-analysis of data from 1,853,610 adults. Br. J. Sports Med..

[18] Lee S.W., Lee J., Moon S.Y., Jin H.Y., Yang J.M., Ogino S., Song M., Hong S.H., Ghayda R.A., Kronbichler A. (2021). Physical activity and the risk of SARS-CoV-2 infection, severe COVID-19 illness and COVID-19 related mortality in South Korea: A nationwide cohort study. Br. J. Sports Med..

[19] Steenkamp L., Saggers R.T., Bandini R., Stranges S., Choi Y.-H., Thornton J.S., Hendrie S., Patel D., Rabinowitz S., Patricios J. (2022). Small steps, strong shield: Directly measured, moderate physical activity in 65,361 adults is associated with significant protective effects from severe COVID-19 outcomes. Br. J. Sports Med..

[20] Ashra F., Jen H.-J., Liu D., Lee T.-Y., Pien L.-C., Chen R., Lin H.-C., Chou K.-R. (2023). Effectiveness of respiratory rehabilitation in patients with COVID-19: A meta-analysis. J. Clin. Nurs..

[21] Filgueira T.O., Castoldi A., Santos L.E.R., de Amorim G.J., de Sousa Fernandes M.S., Anastácio W.L.D.N., Campos E.Z., Santos T.M., Souto F.O. (2021). The Relevance of a Physical Active Lifestyle and Physical Fitness on Immune Defense: Mitigating Disease Burden, With Focus on COVID-19 Consequences. Front. Immunol..

[22] Pinto A.J., Bergouignan A., Dempsey P.C., Roschel H., Owen N., Gualano B., Dunstan D.W. (2023). Physiology of sedentary behavior. Physiol. Rev..

[23] Mehandru S., Merad M. (2022). Pathological sequelae of long-haul COVID. Nat. Immunol..

[24] Feter N., Caputo E.L., Delpino F.M., Leite J.S., da Silva L.S., de Almeida Paz I., Santos Rocha J.Q., Vieira Y.P., Schröeder N., da Silva C.N. (2023). Physical activity and long COVID: Findings from the Prospective Study About Mental and Physical Health in Adults cohort. Public Health.

[25] Rocha J.Q.S., Caputo E.L., Vieira Y.P., Afonso M.D.S., Duro S.M.S., de Oliveira Saes M. (2023). Physical activity status prevents symptoms of long covid: Sulcovid-19 survey. BMC Sports Sci. Med. Rehabil..

[26] Wang S., Li Y., Yue Y., Yuan C., Kang J.-H., Chavarro J.E., Bhupathiraju S.N., Roberts A.L. (2023). Adherence to Healthy Lifestyle Prior to Infection and Risk of Post-COVID-19 Condition. JAMA Intern. Med..

[27] Massey D., Saydah S., Adamson B., Lincoln A., Aukerman D.F., Berke E.M., Sikka R., Krumholz H.M. (2023). Prevalence of COVID-19 and long covid in collegiate student athletes from spring 2020 to fall 2021: A retrospective survey. BMC Infect. Dis..

[28] Neuhann F., Buess M., Wolff A., Pusch L., Kossow A., Winkler M., Demir J., Beyé M., Wiesmüller G.A., Nießen J. (2020). Entwicklung einer Software zur Unterstützung der Prozesse im Gesundheitsamt der Stadt Köln in der SARS-CoV-2-Pandemie, Digitales Kontaktmanagement (DiKoMa). Epidemiol. Bull..

[29] Betsch C., Wieler L., Bosnjak M., Ramharter M., Stollorz V., Omer S., Korn L., Sprengholz P., Felgendreff L., Eitze S. (2020). Germany COVID-19 Snapshot MOnitoring (COSMO Germany): Monitoring Knowledge, Risk Perceptions, Preventive Behaviours, and Public Trust in the Current Coronavirus Outbreak in Germany. PsychArchives.

[30] Unipark Online Umfrage | Online Umfrage Erstellen. https://www.unipark.com/.

[31] Joisten C., Kossow A., Book J., Broichhaus L., Daum M., Eisenburger N., Fabrice A., Feddern S., Gehlhar A., Graf A.C. (2021). How to manage quarantine-adherence, psychosocial consequences, coping strategies and lifestyle of patients with COVID-19 and their confirmed contacts: Study protocol of the CoCo-Fakt surveillance study, Cologne, Germany. BMJ Open.

[32] Lampert T., Kroll L., Müters S., Stolzenberg H. (2013). Messung des sozioökonomischen Status in der Studie zur Gesundheit Erwachsener in Deutschland (DEGS1). Bundesgesundheitsblatt Gesundheitsforsch. Gesundheitsschutz.

[33] World Health Organization (2000). Obesity: Preventing and managing the global epidemic. Report of a WHO consultation. World Health Organ. Tech. Rep. Ser..

[34] World Health Organization (2021). Therapeutics and COVID-19: Living Guideline, 31 March 2021.

[35] Feldt T., Jensen B., Guggemos W., Heim K., Lübbert C., Mikolajewska A., Niebank M., Pfäfflin F., Rothfuss K., Schmiedel S. (2021). Hinweise zu Erkennung, Diagnostik und Therapie von Patienten mit COVID-19 2020.

[36] Lopez-Leon S., Wegman-Ostrosky T., Perelman C., Sepulveda R., Rebolledo P.A., Cuapio A., Villapol S. (2021). More than 50 long-term effects of COVID-19: A systematic review and meta-analysis. Sci. Rep..

[37] Graf C., Schlepper S., Bauer C., Ferrari N., Frank S., Gartner L., Gehring S., Henke R., Lehmacher W., Steffen H.-M. (2016). Feasibility and acceptance of exercise recommendations (10,000 steps a day) within routine German health check (Check-Up 35/GOÄ29)-study protocol. Pilot Feasibility Stud..

[38] Ainsworth B.E., Haskell W.L., Herrmann S.D., Meckes N., Bassett D.R., Tudor-Locke C., Greer J.L., Vezina J., Whitt-Glover M.C., Leon A.S. (2011). 2011 Compendium of Physical Activities: A second update of codes and MET values. Med. Sci. Sports Exerc..

[39] Natarajan A., Shetty A., Delanerolle G., Zeng Y., Zhang Y., Raymont V., Rathod S., Halabi S., Elliot K., Shi J.Q. (2023). A systematic review and meta-analysis of long COVID symptoms. Syst. Rev..

[40] Vollrath S., Bizjak D.A., Zorn J., Matits L., Jerg A., Munk M., Schulz S.V.W., Kirsten J., Schellenberg J., Steinacker J.M. (2022). Recovery of performance and persistent symptoms in athletes after COVID-19. PLoS ONE.

[41] Hull J.H., Wootten M., Moghal M., Heron N., Martin R., Walsted E.S., Biswas A., Loosemore M., Elliott N., Ranson C. (2022). Clinical patterns, recovery time and prolonged impact of COVID-19 illness in international athletes: The UK experience. Br. J. Sports Med..

[42] Schultheiß C., Willscher E., Paschold L., Gottschick C., Klee B., Henkes S.-S., Bosurgi L., Dutzmann J., Sedding D., Frese T. (2022). The IL-1β, IL-6, and TNF cytokine triad is associated with post-acute sequelae of COVID-19. Cell Rep. Med..

[43] Cervia-Hasler C., Brüningk S.C., Hoch T., Fan B., Muzio G., Thompson R.C., Ceglarek L., Meledin R., Westermann P., Emmenegger M. (2024). Persistent complement dysregulation with signs of thromboinflammation in active Long Covid. Science.

[44] Peluso M.J., Abdel-Mohsen M., Henrich T.J., Roan N.R. (2024). Systems analysis of innate and adaptive immunity in Long COVID. Semin. Immunol..

[45] Saito S., Shahbaz S., Osman M., Redmond D., Bozorgmehr N., Rosychuk R.J., Lam G., Sligl W., Cohen Tervaert J.W., Elahi S. (2024). Diverse immunological dysregulation, chronic inflammation, and impaired erythropoiesis in long COVID patients with chronic fatigue syndrome. J. Autoimmun..

[46] Simpson R.J., Kunz H., Agha N., Graff R. (2015). Exercise and the Regulation of Immune Functions. Prog. Mol. Biol. Transl. Sci..

[47] Wunsch K., Kienberger K., Niessner C. (2022). Changes in Physical Activity Patterns Due to the COVID-19 Pandemic: A Systematic Review and Meta-Analysis. Int. J. Environ. Res. Public Health.

[48] Natilli M., Rossi A., Trecroci A., Cavaggioni L., Merati G., Formenti D. (2022). The long-tail effect of the COVID-19 lockdown on Italians’ quality of life, sleep and physical activity. Sci. Data.

[49] Martínez-de-Quel Ó., Suárez-Iglesias D., López-Flores M., Pérez C.A. (2021). Physical activity, dietary habits and sleep quality before and during COVID-19 lockdown: A longitudinal study. Appetite.

[50] Leale I., Giustino V., Trapani P., Alonge P., Rini N., Cutrò I., Leone O., Torrente A., Lupica A., Palma A. (2024). Physical Activity in Patients with Neuromuscular Disease Three Years after COVID-19, a Longitudinal Survey: The After-Effects of the Quarantine and the Benefits of a Return to a Healthier Life-Style. J. Clin. Med..

[51] Palstam A., Seljelid J., Persson H.C., Sunnerhagen K.S. (2024). Physical activity, acute severity and long-term consequences of COVID-19: An 18-month follow-up survey based on a Swedish national cohort. BMJ Open.

[52] Richter A., Schienkiewitz A., Starker A., Krug S., Domanska O., Kuhnert R., Loss J., Mensink G.B.M. (2021). Erratum: Health-promoting behaviour among adults in Germany—Results from GEDA 2019/2020-EHIS. J. Health Monit..

