# Supplementary material for: Associations Between Pre-Quarantine Exercise and Persistent Symptoms After SARS-CoV-2 Infection
Source: Sports (Basel). 2026 Jul 9;14(7):293. doi: 10.3390/sports14070293 (PMC13417650; doi:10.3390/sports14070293)
Supplement: Supplementary file 1 [file sports-14-00293-s001.zip › sports-4363652-supplementary.pdf]

Supplementary File S1
Survey of the second and third wave of the CoCo-Fakt study

Online survey

Information on the study (sub-projects A, B, G)

CoCo-Fakt – Cologne-Corona-Beratung und Unterstützung Für Index- und KontAKt-Personen während der Quarantäne-ZeiT (Cologne-Corona counseling and support for index patients and contacts during the quarantine period)

Thank you for your interest in this survey. Following the provided link, you have arrived at our survey on the online platform. Your participation in this study is voluntary. All information that you provide is anonymous. Only your email address was used to process this online procedure, and it will be irretrievably deleted following your participation. Still, before you decide on whether to participate, you should understand why this survey is being conducted and what is involved. Please take the time to carefully read the following information and decide whether or not you want to participate. If you have any questions about the survey, you can always contact the persons mentioned in the linked privacy policy. They will answer your questions as soon as possible.

Dear study participant,

Since the first infections with the COVID-19 virus were identified in Wuhan in December 2019, it has spread worldwide within a very short period of time and led to considerable restrictions in everyone's lives. To date, little is known about the care and course of the disease during periods of domestic isolation or quarantine. This includes not only the patients themselves but also first-order contacted persons designated as contacts in line with the Infection Protection Act. Given the duration of the restrictive measures to which people may be subjected, it is important to investigate the approaches and creative solutions relevant to people's personal situations, as well as to elaborate on which factors have an impact on the course of the disease.
Therefore, the purpose of this online survey is to gain knowledge about aspects of COVID-19 infections and/or the associated quarantine/domestic isolation. Understanding reactive measures' consequences on both the medical and psychosocial levels is necessary—even now that some time has passed since the quarantine—to generate recommendations for how to handle them in the coming months.
This questionnaire features questions regarding your current situation, how you're dealing with it, your overall lifestyle, your personal assessment of the situation (as well as that of your children) and, if applicable, your pregnancy. It was developed on the basis of the World Health Organization's COVID-19 Snapshot Monitoring (COSMO) survey. Responding to all survey questions will take about 30 minutes.

Privacy Policy
The city's health department collects personal data from you for the purpose of scientific monitoring. The protection of your personal data is a central concern. Accordingly, we feel obliged to comply with the legal requirements, particularly the European Basic Data Protection Regulation, the Data Protection Act of the State of North Rhine-Westphalia, the Infection Protection Act and the Higher Education Act of the State of North Rhine-Westphalia.

Please follow this link to participate in the survey in Turkish:
Anketin türkce versiyonuna katılmak istiyorum.

❑ I consent to participate in this study.

Personal background

First, there are some basic personal questions.

1) **How old are you?** _______________ years old

2) **What is your sex?**

- o Female
- o Male
- o Other

3) **Which language do you primarily speak at home?** __________________

4) **What is your postcode?** __________________

5) **What is your highest level of education?**
Please match any qualifications obtained abroad with the equivalent German qualifications.

- o A-levels, general or subject-specific university entrance qualification/EOS
- o Technical college entrance qualification/technical secondary school
- o Intermediate school leaving certificate/Realschule/mittlere Reife/POS or Year 10 (before 1965: Year 8)
- o Primary school
- o School completed without a qualification
- o No qualification/pupil
- o Other school qualification (e.g., obtained abroad)
- o How many years of schooling have you completed? _________________

6) **What is your highest vocational qualification?**
Please match any qualifications obtained abroad with the equivalent German qualifications.

- o University or college
- o University of applied sciences, engineering school
- o Technical college (e.g., master craftman's school, technical college, vocational or technical academy)
- o Training at a vocational school or commercial school (state-run vocational training)
- o Apprenticeship (in-company vocational training)
- o No qualification or still in vocational training (e.g., student, trainee, vocational preparation year, intern)
- o No vocational qualification and not in training
- o Other qualification

7) **What is your employment status?**

- o Working full-time
- o Working part-time
- o Occasionally or irregularly employed
- o Not employed

8) **What is your main job title?**

- o Worker → AR
- o Employee → AN
- o Civil servant (including conscripted soldiers and judges) → BE
- o Freelancer or self-employed person (including farmers) → BPSF1
- o Family member helping out
- o Other

AR: What type of worker are you?

- o Unskilled worker
- o Semi-skilled worker
- o Skilled or specialist worker
- o Foreman/forewoman, team leader
- o Master craftsman/craftswoman, site supervisor, brigade leader

AN: What type of employee are you?

- o Execute tasks according to general instructions (e.g., cashier, data entry clerk, porter)
- o Carry out qualified work according to instructions (e.g., clerk, specialist sales assistant, technical draughtsman)
- o Perform independent work in a position of responsibility, including specialist responsibility for staff (e.g., research assistant, authorised signatory, head of department)
- o Engage in comprehensive management duties and decision-making powers (e.g., director, managing director, board member)

BE: Which career group do you belong to?
Civil servant in…

- o lower service (up to and including senior administrative officer)
- o middle service (from assistant up to and including chief secretary or administrative inspector)
- o senior service (from inspector up to and including senior administrative officer)
- o senior service (from councillor upwards; e.g., judge, professor)

BPSF1: What type of self-employed person are you?

- o Self-employed farmer or cooperative farmer → BPSF2
- o Academic in a liberal profession (e.g., doctor, lawyer, tax adviser) → BPSF3
- o Self-employed in retail, trade, crafts, industry or services (including sole traders and PGH members) → BPSF3

BPSF2: Do you own, or have you owned, agricultural land (and if so, how much), or are you/were you a cooperative farmer (formerly LPG)?

- o Agricultural land, less than 10 ha
- o Agricultural land, 10 ha or more

- Cooperative farmer (formerly LPG)

BPSF3: How many employees do/did you have?

- No other employees
- 1–4 employees
- 5 or more employees
- PGH member (only for BPFS1; e.g., trade)

Health status

---

9) What is your height? __________ cm

10) What is your weight? ____________ kg

11) Do you have any chronic diseases?

- Yes
- No
- I don’t know

12) Do you have any of the following diseases?
You can select multiple answers.

- Pulmonary disease → LU
- Cardiovascular disease (e.g., high blood pressure, coronary heart disease) → HE
- Neurological disorders (e.g., stroke, multiple sclerosis, headaches) → NE
- Musculoskeletal disorders (e.g., osteoarthritis)
- Metabolic disease (e.g., diabetes, thyroid disorder) → SW
- Chronic gastrointestinal disease → MD
- Chronic kidney problems or kidney failure
- Mental health conditions → PS
- Dermatological disease → HA
- Cancer (if so, which type?) ____________________
- Allergies, not including allergic asthma (e.g., hay fever, allergic reactions affecting the eyes or skin, food allergies)
- Autoimmune disease (e.g., HIV, rheumatoid arthritis) → AI
- Other: _______________

LU: What pulmonary disease(s) do you have?
You can select multiple answers.

- Asthma, including allergic asthma
- Chronic bronchitis, chronic obstructive pulmonary disease (COPD), pulmonary emphysema
- Other: _______________

Has your treatment changed because of the coronavirus pandemic?
You can select multiple answers.

- No
- Yes, change in medication
- Yes, video or telephone consultations instead of appointments (e.g., at hospitals or doctors' offices)
- Yes, lack of support from close relatives
- Yes, medical staff are more stressed
- Yes, cancellation of operations or diagnostic procedures
- Yes, cancellation of regular appointments
- Yes, other: __________________
- I don't know (e.g., initial diagnosis after start of pandemic in March 2020)

Has your treatment changed during your quarantine period?
You can select multiple answers.

- No
- Yes, video or telephone consultations with a medical or psychological service
- Yes, cancellation or postponement of treatments (e.g., chemotherapy, radiotherapy, psychotherapy)
- Yes, treatment at a different location than usual (e.g., dialysis)
- Yes, other: __________________
- I don't know (e.g., initial diagnosis after start of quarantine period)

Did you require medical treatment unrelated to COVID-19 during the quarantine period?

- No
- Yes, due to acute symptoms. What symptoms? __________________
- Yes, due to a planned treatment. What treatment? __________________
- Yes, other: ___________________

Was the treatment at your usual healthcare facility?

- Yes
- No. Where did you receive treatment? __________________

How did you get to the treatment?

- By car (personal vehicle)
- By foot
- By ambulance
- By bicycle
- Other: __________________

How did you find the treatment during your quarantine period relative to your other treatments?

- o Much worse
- o
- o
- o
- o
- o Much better

HE: What cardiovascular disease(s) do you have?

You can select multiple answers.

- o Heart attack, coronary heart disease
- o Heart failure
- o Arrhythmia
- o High blood pressure/hypertension
- o Dyslipidaemia

Has your treatment changed because of the coronavirus pandemic?

You can select multiple answers.

- o No
- o Yes, change in medication
- o Yes, video or telephone consultations instead of appointments (e.g., at hospitals or doctors' offices)
- o Yes, lack of support from close relatives
- o Yes, medical staff are more stressed
- o Yes, cancellation of operations or diagnostic procedures
- o Yes, cancellation of regular appointments
- o Yes, other: ___________________
- o I don't know (e.g., initial diagnosis after start of pandemic in March 2020)

Has your treatment changed during your quarantine period?

You can select multiple answers.

- o No
- o Yes, video or telephone consultations with a medical or psychological service
- o Yes, cancellation or postponement of treatments (e.g., chemotherapy, radiotherapy, psychotherapy)
- o Yes, treatment at a different location than usual (e.g., dialysis)
- o Yes, other: ___________________
- o I don't know (e.g., initial diagnosis after start of quarantine period)

Did you require medical treatment unrelated to COVID-19 during the quarantine period?

- o No
- o Yes, due to acute symptoms. What symptoms? __________________
- o Yes, due to a planned treatment. What treatment? __________________
- o Yes, other: ___________________

Was the treatment at your usual healthcare facility?

- o Yes
- o No. Where did you receive treatment? __________________

How did you get to the treatment?

- o By car (personal vehicle)
- o By foot
- o By ambulance
- o By bicycle
- o Other: __________________

How did you find the treatment during your quarantine period relative to your other treatments?

- o Much worse
- o
- o
- o
- o
- o Much better

NE: What neurological disorder(s) do you have?
You can select multiple answers.

- o Stroke
- o Multiple sclerosis
- o Headaches (e.g., migraine, cluster headache)
- o Epilepsy
- o Other: _______________

Has your treatment changed because of the coronavirus pandemic?
You can select multiple answers.

- o No
- o Yes, change in medication
- o Yes, video or telephone consultations instead of appointments (e.g., at hospitals or doctors' offices)
- o Yes, lack of support from close relatives
- o Yes, medical staff are more stressed
- o Yes, cancellation of operations or diagnostic procedures

- Yes, cancellation of regular appointments
- Yes, other: __________________
- I don't know (e.g., initial diagnosis after start of pandemic in March 2020)

Has your treatment changed during your quarantine period?
You can select multiple answers.

- No
- Yes, video or telephone consultations with a medical or psychological service
- Yes, cancellation or postponement of treatments (e.g., chemotherapy, radiotherapy, psychotherapy)
- Yes, treatment at a different location than usual (e.g., dialysis)
- Yes, other: __________________
- I don't know (e.g., initial diagnosis after start of quarantine period)

Did you require medical treatment unrelated to COVID-19 during the quarantine period?

- No
- Yes, due to acute symptoms. What symptoms? __________________
- Yes, due to a planned treatment. What treatment? __________________
- Yes, other: ___________________

Was the treatment at your usual healthcare facility?

- Yes
- No. Where did you receive treatment? __________________

How did you get to the treatment?

- By car (personal vehicle)
- By foot
- By ambulance
- By bicycle
- Other: __________________

How did you find the treatment during your quarantine period relative to your other treatments?

- Much worse
-
-
-
-
- Much better

SW: What metabolic disease(s) do you have?

You can select multiple answers.

- Diabetes, type 1 or type 2, not including gestational diabetes
- Thyroid disorder, including Hashimoto’s
- Other: _______________

Has your treatment changed because of the coronavirus pandemic?

You can select multiple answers.

- No
- Yes, change in medication
- Yes, video or telephone consultations instead of appointments (e.g., at hospitals or doctors’ offices)
- Yes, lack of support from close relatives
- Yes, medical staff are more stressed
- Yes, cancellation of operations or diagnostic procedures
- Yes, cancellation of regular appointments
- Yes, other: __________________
- I don’t know (e.g., initial diagnosis after start of pandemic in March 2020)

Has your treatment changed during your quarantine period?

You can select multiple answers.

- No
- Yes, video or telephone consultations with a medical or psychological service
- Yes, cancellation or postponement of treatments (e.g., chemotherapy, radiotherapy, psychotherapy)
- Yes, treatment at a different location than usual (e.g., dialysis)
- Yes, other: __________________
- I don’t know (e.g., initial diagnosis after start of quarantine period)

Did you require medical treatment unrelated to COVID-19 during the quarantine period?

- No
- Yes, due to acute symptoms. What symptoms? __________________
- Yes, due to a planned treatment. What treatment? __________________
- Yes, other: ___________________

Was the treatment at your usual healthcare facility?

- Yes
- No. Where did you receive treatment? __________________

How did you get to the treatment?

- By car (personal vehicle)
- By foot
- By ambulance
- By bicycle
- Other: __________________

How did you find the treatment during your quarantine period relative to your other treatments?

- Much worse
-
-
-
-
- Much better

MD: What chronic gastrointestinal disorder(s) do you have?

You can select multiple answers.

- Chronic liver diseases
- Inflammatory bowel disease (e.g., Crohn’s disease, ulcerative colitis)
- Irritable bowel syndrome
- Chronic gastritis
- Other: _______________

Has your treatment changed because of the coronavirus pandemic?

You can select multiple answers.

- No
- Yes, change in medication
- Yes, video or telephone consultations instead of appointments (e.g., at hospitals or doctors’ offices)
- Yes, lack of support from close relatives
- Yes, medical staff are more stressed
- Yes, cancellation of operations or diagnostic procedures
- Yes, cancellation of regular appointments
- Yes, other: __________________
- I don’t know (e.g., initial diagnosis after start of pandemic in March 2020)

Has your treatment changed during your quarantine period?

You can select multiple answers.

- No
- Yes, video or telephone consultations with a medical or psychological service
- Yes, cancellation or postponement of treatments (e.g., chemotherapy, radiotherapy, psychotherapy)
- Yes, treatment at a different location than usual (e.g., dialysis)

- Yes, other: __________________
- I don’t know (e.g., initial diagnosis after start of quarantine period)

Did you require medical treatment unrelated to COVID-19 during the quarantine period?

- No
- Yes, due to acute symptoms. What symptoms? __________________
- Yes, due to a planned treatment. What treatment? __________________
- Yes, other: ___________________

Was the treatment at your usual healthcare facility?

- Yes
- No. Where did you receive treatment? __________________

How did you get to the treatment?

- By car (personal vehicle)
- By foot
- By ambulance
- By bicycle
- Other: __________________

How did you find the treatment during your quarantine period relative to your other treatments?

- Much worse
-
-
-
-
- Much better

PS: What mental health condition(s) do you have?

You can select multiple answers.

- Depression
- Bipolar affective disorder
- Anxiety disorder
- Borderline
- Eating disorder
- Personality disorder
- Post-traumatic stress disorder
- Schizoaffective disorder
- Other: _______________

Has your treatment changed because of the coronavirus pandemic?

You can select multiple answers.

- No
- Yes, change in medication
- Yes, video or telephone consultations instead of appointments (e.g., at hospitals or doctors' offices)
- Yes, lack of support from close relatives
- Yes, medical staff are more stressed
- Yes, cancellation of operations or diagnostic procedures
- Yes, cancellation of regular appointments
- Yes, other: __________________
- I don't know (e.g., initial diagnosis after start of pandemic in March 2020)

Has your treatment changed during your quarantine period?

You can select multiple answers.

- No
- Yes, video or telephone consultations with a medical or psychological service
- Yes, cancellation or postponement of treatments (e.g., chemotherapy, radiotherapy, psychotherapy)
- Yes, treatment at a different location than usual (e.g., dialysis)
- Yes, other: __________________
- I don't know (e.g., initial diagnosis after start of quarantine period)

Did you require medical treatment unrelated to COVID-19 during the quarantine period?

- No
- Yes, due to acute symptoms. What symptoms? __________________
- Yes, due to a planned treatment. What treatment? __________________
- Yes, other: ___________________

Was the treatment at your usual healthcare facility?

- Yes
- No. Where did you receive treatment? __________________

How did you get to the treatment?

- By car (personal vehicle)
- By foot
- By ambulance
- By bicycle
- Other: __________________

How did you find the treatment during your quarantine period relative to your other treatments?

- Much worse
-
-
-
-
- Much better

GP: What dermatological disease(s) do you have?

You can select multiple answers.

- Psoriasis
- Atopic dermatitis
- Other: _______________

Has your treatment changed because of the coronavirus pandemic?

You can select multiple answers.

- No
- Yes, change in medication
- Yes, video or telephone consultations instead of appointments (e.g., at hospitals or doctors' offices)
- Yes, lack of support from close relatives
- Yes, medical staff are more stressed
- Yes, cancellation of operations or diagnostic procedures
- Yes, cancellation of regular appointments
- Yes, other: __________________
- I don't know (e.g., initial diagnosis after start of pandemic in March 2020)

Has your treatment changed during your quarantine period?

You can select multiple answers.

- No
- Yes, video or telephone consultations with a medical or psychological service
- Yes, cancellation or postponement of treatments (e.g., chemotherapy, radiotherapy, psychotherapy)
- Yes, treatment at a different location than usual (e.g., dialysis)
- Yes, other: __________________
- I don't know (e.g., initial diagnosis after start of quarantine period)

Did you require medical treatment unrelated to COVID-19 during the quarantine period?

- No
- Yes, due to acute symptoms. What symptoms? __________________
- Yes, due to a planned treatment. What treatment? __________________
- Yes, other: ___________________

Was the treatment at your usual healthcare facility?

- Yes
- No. Where did you receive treatment? __________________

How did you get to the treatment?

- By car (personal vehicle)
- By foot
- By ambulance
- By bicycle
- Other: __________________

How did you find the treatment during your quarantine period relative to your other treatments?

- Much worse
-
-
-
-
- Much better

AI: What autoimmune disease(s) do you have?

You can select multiple answers.

- HIV
- Rheumatoid arthritis
- Other: _______________

Has your treatment changed because of the coronavirus pandemic?

You can select multiple answers.

- No
- Yes, change in medication
- Yes, video or telephone consultations instead of appointments (e.g., at hospitals or doctors' offices)
- Yes, lack of support from close relatives
- Yes, medical staff are more stressed
- Yes, cancellation of operations or diagnostic procedures
- Yes, cancellation of regular appointments
- Yes, other: __________________
- I don't know (e.g., initial diagnosis after start of pandemic in March 2020)

Has your treatment changed during your quarantine period?

You can select multiple answers.

- o No
- o Yes, video or telephone consultations with a medical or psychological service
- o Yes, cancellation or postponement of treatments (e.g., chemotherapy, radiotherapy, psychotherapy)
- o Yes, treatment at a different location than usual (e.g., dialysis)
- o Yes, other: __________________
- o I don't know (e.g., initial diagnosis after start of quarantine period)

Did you require medical treatment unrelated to COVID-19 during the quarantine period?

- o No
- o Yes, due to acute symptoms. What symptoms? __________________
- o Yes, due to a planned treatment. What treatment? __________________
- o Yes, other: ___________________

Was the treatment at your usual healthcare facility?

- o Yes
- o No. Where did you receive treatment? __________________

How did you get to the treatment?

- o By car (personal vehicle)
- o By foot
- o By ambulance
- o By bicycle
- o Other: __________________

How did you find the treatment during your quarantine period relative to your other treatments?

- o Much worse
- o
- o
- o
- o
- o Much better

Further personal information

13) Are you living in a relationship or partnership (including marriage)?

- o Yes
- o No

14) **Apart from yourself, how many adults live at your address? ___________**

15) **Do you have children under the age of 16?**
Children aged 16 and over who have been in quarantine are asked to complete the survey independently

- o Yes
- o No

16) **If yes, how many children under the age of 16 do you have? ________________**

17) **Please indicate the extent to which you agree with the following statements regarding your family:**

	Completely disagree	Slightly disagree	Slightly agree	Completely agree
In our family everybody cares about each other's worries	O	O	O	O
We all get along really well	O	O	O	O
Whatever we do at home, we do it with enthusiasm	O	O	O	O
In our family, everyone feels that they are listened to and taken seriously	O	O	O	O

Would you like some support in dealing with your child(ren) during the coronavirus crisis?

- o No, never
- o Yes, sometime
- o Yes, often
- o Yes, always

In which areas would you like support?
You can select multiple answers.

- o Dealing with my child(ren)'s feelings and moods
- o Dealing with my child(ren)'s behaviour
- o Dealing with my child(ren)'s academic demands
- o Ensuring that my child(ren) follow(s) government guidelines
- o Dealing with family relationships
- o My child(ren) returning from isolation
- o Other: __________________

How would you like to receive this support?
You can select multiple answers.

- Written online material (e.g., guides)
- Online videos
- TV programmes
- Podcasts
- Telephone helpline
- Online helpline
- Personal support from other parents (online)
- Support from friends, acquaintances or family members
- Personal support from experts (online or by phone)
- Face-to-face consultation with an expert
- Self-help group for parents (online)
- Support from school/teachers
- Other: __________________

Housing situation

18) Does your house/apartment have a garden or balcony?

- A garden
- A balcony
- Both
- Neither

19) How many rooms does your household have?
Note: Kitchens, hallways and bathrooms do not count as rooms.

- 1
- 2 or 2.5
- 3 or 3.5
- 4 or 4.5
- 5 or 5.5
- 6 or 6.5
- 7 or more rooms

20) Do you live alone?
Note: This refers to the household in which you spent most of your quarantine period.

- Yes
- No

21) During your quarantine period, did any other members of your household contract COVID-19, or were they in quarantine due to a positive test result of their own?

- Yes
- No

22) **Is it generally possible for you to stay in a room on your own during quarantine?**

- o Yes
- o No

23) **Did you have to isolate yourself from your partner, children or family during quarantine?**

- o Yes, in a shared apartment
- o Yes, in a separate apartment
- o No, because: _______________

24) **How were your relationships with the people with whom you were in quarantine?**

- o Very good
- o Good
- o Slightly good
- o Slightly poor
- o Poor
- o Very poor

Vaccination status

25) **Have you already received a COVID-19 vaccination?**

- o Yes, I received one dose of the vaccine.
- o Yes, I received two doses of the vaccine as of _________ days ago.
- o Yes, I have been vaccinated and have already recovered from the infection.
- o No, I haven't been vaccinated yet, but I would like to be vaccinated.
- o No, and I do not wish to be vaccinated.

Your quarantine situation

26) **Why did you need to quarantine?**
If you had to quarantine more than once, please refer to your most recent quarantine.

- o I tested positive for COVID-19 with a PCR test
- o I tested positive for COVID-19 *only* via a rapid test
- o I was a contact person
- o I was a contact person several times
- o I was a contact person and subsequently tested positive for COVID-19
- o I was a travel returnee
- o Other: _______________
- o I don't know

Your quarantine situation (continued)

27) I got tested because…

- o … I had symptoms.
- o … I had been in contact with someone who tested positive.
- o … my employer/school/daycare required it.
- o Other: ______________________

28) Did you have to quarantine more than once?

- o Yes
- o No

29) How long ago was your last quarantine? _____ weeks

30) If you quarantined only once: For how many days did you need to quarantine? _________ days

31) If you quarantined more than once: For how many days did you need to quarantine?

First quarantine: ______ days
Second quarantine: ______ days
Third quarantine: ______ days

Infected persons

32) Did you have a virus mutation?

- o No
- o Alpha (English variant B 1.1.7)
- o Beta (South African variant B.1.351)
- o Gamma (Brazilian variant P.1)
- o Delta (Indian variant B.1.617.2)
- o Combined variant
- o I don't know
- o Other: _______________

33) How would you describe the course of your illness?
You can select multiple answers.

- o Completely symptom-free
- o Mild symptoms. On how many days? ______
- o Significant symptoms. On how many days? _______
- o Severe feeling of illness. On how many days? _______
- o Disease-related anxiety
- o Long-term symptoms (post-COVID-19 conditions/>4 weeks)
- o Other: _______________

34) Which symptoms did you experience in the first two weeks after your positive test?

	Did not occur	Mild symptoms	Moderate symptoms	Severe symptoms
Fever (>38°C), for how many days? _________	O	O	O	O
Cough, for how many days? _________	O	O	O	O
Loss of appetite, for how many days? _________	O	O	O	O
Fatigue, for how many days? _________	O	O	O	O
Mild flu symptoms (e.g., stuffy nose, sore throat), for how many days? _________	O	O	O	O
Muscle exhaustion, for how many days? _________	O	O	O	O
Diarrhoea, for how many days? _________	O	O	O	O
Dyspnoea (shortness of breath), for how many days? _________	O	O	O	O
Headache, for how many days? _________	O	O	O	O

35) **What long-term symptoms are you experiencing/did you experience after your quarantine?**
This refers to symptoms that are/were still present at least four weeks after the positive test result, or those that newly appeared at least four weeks after the test.

- o Fatigue
- o Cognitive impairment (e.g., 'brain fog', loss of concentration, memory issues)
- o Loss of taste
- o Loss of smell
- o Respiratory symptoms (e.g., breathlessness, shortness of breath)
- o Psychological or psychiatric symptoms (e.g., depression, anxiety, post-traumatic stress disorder)
- o Sleep disturbance
- o Muscular exhaustion
- o Headaches
- o Hair loss
- o Other: ___________________

36) **How many week(s) after the positive test did you experience these long-term symptoms?**

**Fatigue**	**No symptoms**	**Mild symptoms**	**Moderate symptoms**	**Severe symptoms**
Up to 4 weeks	O	O	O	O
4 to 8 weeks	O	O	O	O
More than 8 weeks, less than 3 months	O	O	O	O
3 months or more	O	O	O	O

37) **How many week(s) after the positive test did you experience these long-term symptoms?**

**Cognitive impairment**	**No symptoms**	**Mild symptoms**	**Moderate symptoms**	**Severe symptoms**
Up to 4 weeks	O	O	O	O
4 to 8 weeks	O	O	O	O
More than 8 weeks, less than 3 months	O	O	O	O
3 months or more	O	O	O	O

38) **How many week(s) after the positive test did you experience these long-term symptoms?**

**Loss of taste**	**No symptoms**	**Mild symptoms**	**Moderate symptoms**	**Severe symptoms**
Up to 4 weeks	O	O	O	O
4 to 8 weeks	O	O	O	O
More than 8 weeks, less than 3 months	O	O	O	O
3 months or more	O	O	O	O

39) How many week(s) after the positive test did you experience these long-term symptoms?

Loss of smell	No symptoms	Mild symptoms	Moderate symptoms	Severe symptoms
Up to 4 weeks	O	O	O	O
4 to 8 weeks	O	O	O	O
More than 8 weeks, less than 3 months	O	O	O	O
3 months or more	O	O	O	O

40) How many week(s) after the positive test did you experience these long-term symptoms?

Respiratory symptoms	No symptoms	Mild symptoms	Moderate symptoms	Severe symptoms
Up to 4 weeks	O	O	O	O
4 to 8 weeks	O	O	O	O
More than 8 weeks, less than 3 months	O	O	O	O
3 months or more	O	O	O	O

41) How many week(s) after the positive test did you experience these long-term symptoms?

Psychological or psychiatric symptoms	No symptoms	Mild symptoms	Moderate symptoms	Severe symptoms
Up to 4 weeks	O	O	O	O
4 to 8 weeks	O	O	O	O
More than 8 weeks, less than 3 months	O	O	O	O
3 months or more	O	O	O	O

42) How many week(s) after the positive test did you experience these long-term symptoms?

Sleep disturbance	No symptoms	Mild symptoms	Moderate symptoms	Severe symptoms
Up to 4 weeks	O	O	O	O
4 to 8 weeks	O	O	O	O
More than 8 weeks, less than 3 months	O	O	O	O
3 months or more	O	O	O	O

43) **How many week(s) after the positive test did you experience these long-term symptoms?**

**Muscular exhaustion**	**No symptoms**	**Mild symptoms**	**Moderate symptoms**	**Severe symptoms**
Up to 4 weeks	O	O	O	O
4 to 8 weeks	O	O	O	O
More than 8 weeks, less than 3 months	O	O	O	O
3 months or more	O	O	O	O

44) **How many week(s) after the positive test did you experience these long-term symptoms?**

**Headache**	**No symptoms**	**Mild symptoms**	**Moderate symptoms**	**Severe symptoms**
Up to 4 weeks	O	O	O	O
4 to 8 weeks	O	O	O	O
More than 8 weeks, less than 3 months	O	O	O	O
3 months or more	O	O	O	O

45) **How many week(s) after the positive test did you experience these long-term symptoms?**

**Hair loss**	**No symptoms**	**Mild symptoms**	**Moderate symptoms**	**Severe symptoms**
Up to 4 weeks	O	O	O	O
4 to 8 weeks	O	O	O	O
More than 8 weeks, less than 3 months	O	O	O	O
3 months or more	O	O	O	O

46) **How many week(s) after the positive test did you experience these long-term symptoms?**

**Other long-term symptoms**	**No symptoms**	**Mild symptoms**	**Moderate symptoms**	**Severe symptoms**
Up to 4 weeks	O	O	O	O
4 to 8 weeks	O	O	O	O
More than 8 weeks, less than 3 months	O	O	O	O
3 months or more	O	O	O	O

47) How often do the long-term symptoms affect you…

	Never					Always
…in your private life? (e.g., shopping, going for walks, leisure)	O	O	O	O	O	O
... in your professional life?	O	O	O	O	O	O

48) To what extent do/did you feel restricted by the long-term symptoms?

- o Not at all restricted
- o
- o
- o
- o
- o Highly restricted

49) Do you have a suspicion as to how you were infected?

- o Yes
- o No

50) During your quarantine period, did any other members of your household have COVID-19, or were they in quarantine due to a positive test result?

- o Yes
- o No

We would like to ask you about the situation in which you may have been infected or had contact with a coronavirus patient.

51) I may have been infected/had contact with a coronavirus patient in the following situation:
Please identify the situation you think is the most likely to have led to you contracting COVID-19.

- o With family members who live with me
- o With family members who do not live with me
- o With friends or acquaintances (not at a celebration/party)
- o At work
- o At school/university/daycare
- o While receiving medical treatment (e.g., doctor's office, hospital, rehabilitation clinic)

- On public transport (e.g., bus, train, tram)
- While shopping
- During sport (e.g., in a club, in the gym)
- At a party or family celebration with approximately ________ people
- In a restaurant, bar or café
- In a religious institution (e.g., church, mosque)
- Other: _______________

52) Was the person you had contact with wearing a mask?

- No
- Community mask
- Medical face mask
- FFP2 mask
- FFP3 mask
- Other: _______________
- I don't know

53) I was wearing the following protection during the contact:
You can select multiple answers.

- None
- Community mask
- Medical face mask
- FFP2 mask
- FFP3 mask
- Protective gown
- Gloves
- Other: _______________
- I don't know

54) Did the contact take place within enclosed spaces?

- Yes
- No

55) The total duration of the contact was: ________ minutes

56) The distance at which the contact was made was: _________ metres

57) If the contact took place in the workplace: Which of the following applies to you?

- I work as a healthcare professional (medical staff)
- I work as a healthcare professional (nursing staff at nursing home or outpatient nursing service)
- I work in an office with _______________ other people
- I work in the retail sector
- I work in a school/daycare centre
- I work in the following field: _______________

58) If the contact took place on public transport (e.g., bus, train, tram): How often do you use public transport per week?

- 1 to 2 days per week
- 3 to 4 days per week
- 5 to 7 days per week

59) If the contact took place within enclosed spaces: How regularly was the room aired out?

- o Permanently
- o Several times per hour
- o Every 1 to 2 hours
- o Less than every 2 hours
- o There was a ventilation system
- o Not at all
- o I don't know

Knowledge of quarantine recommendations – Part I

In this section, we would like to uncover how you got information about the recommendations for dealing with the quarantine and to what extent you were able to implement them.

60) Please indicate the extent to which you agree with the following statements.

	**Comple tely agree**					**Comple tely disagre e**
1. I think the new coronavirus is dangerous.	O	O	O	O	O	O
2. It was clearly explained to me why I should go into quarantine.	O	O	O	O	O	O
3. It was clearly explained to me how I should behave in quarantine.	O	O	O	O	O	O
4a. I think the quarantine measures are too strict.	O	O	O	O	O	O
4b. I think the quarantine measures are too loose.	O	O	O	O	O	O
5a. When I quarantine, I protect myself.	O	O	O	O	O	O
5b. When I quarantine, I protect other members of my household.	O	O	O	O	O	O
5c. If I quarantine, I will protect our society from the further spread of the coronavirus.	O	O	O	O	O	O
6. People in my professional and social environment expected me to adhere to the quarantine measures.	O	O	O	O	O	O
7. During my quarantine period, I struggled to provide myself with the things I needed for everyday life (e.g., groceries, drug-store items).	O	O	O	O	O	O

Health literacy

61) How easy/difficult is it for you…

	Very easy					***Very difficult***
…to understand the quarantine measures that the health authority has informed you of?	O	O	O	O	O	O
…to understand why it is important to isolate as a contact person?	O	O	O	O	O	O
…to understand what the health authority or your doctor is telling you?	O	O	O	O	O	O
…to follow the instructions from your local health authority on how to behave during quarantine?	O	O	O	O	O	O
…to interpret health advice for quarantine from family members or friends?	O	O	O	O	O	O
…to find information about support services for mental health issues during quarantine?	O	O	O	O	O	O
…to find information on how you should behave during quarantine?	O	O	O	O	O	O
…to assess which activities would be good for you during quarantine or, if applicable, during a COVID-19 infection?	O	O	O	O	O	O
…to find information about activities that could be good for your mental well-being during quarantine (e.g., meditation, physical exercise, Pilates)?	O	O	O	O	O	O
…to apply the information or activities that you found useful for your own situation?	O	O	O	O	O	O
…to understand information in the media about how you can improve your health during quarantine or, if applicable, a COVID-19 infection (e.g., from the internet, newspapers or magazines)?	O	O	O	O	O	O
…to assess whether the information regarding COVID-19 (infections) in the media (e.g., TV, the Internet) is trustworthy?	O	O	O	O	O	O

…to understand health warnings about certain behaviour during quarantine (e.g., smoking, unhealthy eating)?	O	O	O	O	O	O
…to assess which everyday habits are linked to your health and recovery (e.g., drinking frequency, eating habits, exercise)?	O	O	O	O	O	O
…to find information about (COVID-19) symptoms that might apply to you?	O	O	O	O	O	O
…to find information about measures that might be helpful in the context of a COVID-19 infection?	O	O	O	O	O	O
…to find out where you can get professional help if you have COVID-19 (e.g., doctor, pharmacist, psychologist)?	O	O	O	O	O	O
…to assess when you should contact a doctor?	O	O	O	O	O	O
…to find information on what to do in the event of a medical emergency?	O	O	O	O	O	O

Knowledge of quarantine recommendations – Part II

1) <u>Recommendation: Do not leave your apartment/house</u>

Were you aware of this recommendation during your quarantine period?

- o Yes
- o No

	**I fully implemented it**					**I did not implement it at all**
**Did you implement this recommendation?**	O	O	O	O	O	O
	**Not difficult at all**					**Very difficult**
**How difficult did you find implementing this recommendation?**	O	O	O	O	O	O

2) Recommendation: Do not have visitors

Were you aware of this recommendation during your quarantine period?

- o Yes
- o No

	**I fully implemented it**					**I did not implement it at all**
**Did you implement this recommendation?**	O	O	O	O	O	O
	**Not difficult at all**					**Very difficult**
**How difficult did you find implementing this recommendation?**	O	O	O	O	O	O

3) Recommendation: Stay in a single room, separate from other members of your household

Were you aware of this recommendation during your quarantine period?

- o Yes
- o No

	**I fully implemented it**					**I did not implement it at all**
**Did you implement this recommendation?**	O	O	O	O	O	O
	**Not difficult at all**					**Very difficult**
**How difficult did you find implementing this recommendation?**	O	O	O	O	O	O

4) ***Recommendation: Keep a distance of at least 1.5m when in contact with other members of your household***

Were you aware of this recommendation during your quarantine period?

- o Yes
- o No

	**I fully implemented it**					**I did not implement it at all**
**Did you implement this recommendation?**	O	O	O	O	O	O
	**Not difficult at all**					**Very difficult**
**How difficult did you find implementing this recommendation?**	O	O	O	O	O	O

5) ***Recommendation: Wear a face mask when in contact with other members of your household***

Were you aware of this recommendation during your quarantine period?

- o Yes
- o No

	**I fully implemented it**					**I did not implement it at all**
**Did you implement this recommendation?**	O	O	O	O	O	O
	**Not difficult at all**					**Very difficult**
**How difficult did you find implementing this recommendation?**	O	O	O	O	O	O

6) ***Recommendation: Air out all rooms regularly***

Were you aware of this recommendation during your quarantine period?

- Yes
- No

	**I fully implemented it**					**I did not implement it at all**
**Did you implement this recommendation?**	O	O	O	O	O	O
	**Not difficult at all**					**Very difficult**
**How difficult did you find implementing this recommendation?**	O	O	O	O	O	O

7) ***Recommendation: Sneeze into the crook of your arm or a disposable tissue***

Were you aware of this recommendation during your quarantine period?

- Yes
- No

	**I fully implemented it**					**I did not implement it at all**
**Did you implement this recommendation?**	O	O	O	O	O	O
	**Not difficult at all**					**Very difficult**
**How difficult did you find implementing this recommendation?**	O	O	O	O	O	O

8) ***Recommendation: Wash your hands regularly for at least 20 seconds, particularly after blowing your nose or sneezing***

Were you aware of this recommendation during your quarantine period?

- o Yes
- o No

	**I fully implemented it**					**I did not implement it at all**
**Did you implement this recommendation?**	O	O	O	O	O	O
	**Not difficult at all**					**Very difficult**
**How difficult did you find implementing this recommendation?**	O	O	O	O	O	O

62) If you generally found it difficult to implement the quarantine measures: What exactly made the implementation difficult for you?

1) I found the implementation difficult because______________________________
2) I found the implementation difficult because______________________________
3) I found the implementation difficult because______________________________

Mental health

In this section, we would like to uncover how you felt during your quarantine period and how you and others could be supported during this phase.

First, please think back to the time before your quarantine.

63) Please answer the following questions with regard to your situation <u>in the 4 weeks leading up to your quarantine period</u>.

	**Yes**	**No**
a) Have you often been bothered by feeling down, depressed or hopeless?	O	O
b) Have you often been bothered by little interest or pleasure in doing things?	O	O

64) **Please answer the following questions with regard to your situation <u>during your quarantine period</u>.**

	Yes	No
a) Have you often been bothered by feeling down, depressed or hopeless?	O	O
b) Have you often been bothered by little interest or pleasure in doing things?	O	O

65) **Please answer the following questions with regard to your situation <u>over the last 2 weeks</u>.**

	Yes	No
a) Have you often been bothered by feeling down, depressed or hopeless?	O	O
b) Have you often been bothered by little interest or pleasure in doing things?	O	O

66) **Please indicate the degree to which you agree with the following statements with regard to your situation <u>over the last 2 weeks</u>.**

	All the time	Most of the time	Slightly more than half the time	Slightly less than half the time	From time to time	Not at all
I was happy and in a good mood	O	O	O	O	O	O
I felt calm and relaxed	O	O	O	O	O	O
I felt energetic and active	O	O	O	O	O	O
I felt fresh and rested when I woke up	O	O	O	O	O	O
My everyday life was full of things that interest me	O	O	O	O	O	O

67) Please identify the frequency with which you felt or behaved <u>during your quarantine period</u>.

	Not at all or less than 1 day					Always or every day
I felt nervous, anxious or tense	O	O	O	O	O	O
I felt down/depressed	O	O	O	O	O	O
I felt lonely	O	O	O	O	O	O
I thought of the future with hope	O	O	O	O	O	O
I had trouble falling asleep	O	O	O	O	O	O
I had trouble staying asleep	O	O	O	O	O	O
Thoughts of my experiences in the Corona pandemic triggered physical reactions in me, such as sweating, shortness of breath, dizziness or palpitations	O	O	O	O	O	O
I thought about my career with great anxiety	O	O	O	O	O	O

68) How much do/did the following statements apply to you <u>during your quarantine period</u>?

	Fully applicable					Not applicable at all
I received offers of support from family, friends or neighbours.	O	O	O	O	O	O
I had a plan for my daily life in terms of sleep, work or physical activities.	O	O	O	O	O	O
I have discovered activities for myself that make staying at home easier.	O	O	O	O	O	O
I exchanged information with family, friends and acquaintances via digital media.	O	O	O	O	O	O
I was bored.	O	O	O	O	O	O

There was nothing I could do myself to influence the situation positively	O	O	O	O	O	O
There wasn't much change for me compared to before my quarantine period	O	O	O	O	O	O

69) **Please answer the following questions with regard to your experience <u>during your quarantine period</u>.**
Please indicate the extent to which you agree with the following statements.

	Complet ely agree					Completely disagree
I find the ways necessary for me to move on	O	O	O	O	O	O
I know I won't let it get me down	O	O	O	O	O	O
I learn important and useful lessons for my life	O	O	O	O	O	O
I learn ways to deal with it better next time	O	O	O	O	O	O

70) **What helped you most during your quarantine period?**
Please identify up to three elements that you consider most important.

- Social contact (e.g., family, pets, talking to others in the same situation, help from friends or neighbours)
- Taking responsibility for others/caring for others
- A daily routine
- My inner attitude (e.g., optimism, faith, rest, hope for a positive turn in the pandemic, making plans for after quarantine)
- Exercise
- Alcohol/drugs
- Healthy eating
- Sweets or snacks
- My hobbies and activities
- Being outdoors (e.g., on the balcony)
- Avoiding news about the pandemic
- Support from health authorities (e.g., trust in health authorities, phone calls, diary entries)
- Medical care (e.g., contact with GP, hospital stay, supply of medication)
- Ensuring supplies (e.g., food)
- Financial security
- Work/study (e.g., support from employer or colleagues, option to work from home, KRITIS, online lectures)
- No symptoms/long-term effects, or a mild course of the illness in my case
- No symptoms/long-term effects, or a mild course of the illness in the cases of others (e.g., family members, friends, contact persons)

- o Weather
- o Living situation
- o Not having infected anyone else
- o Negative COVID-19 test
- o (Short) quarantine duration
- o Other: ____
- o None

71) What did you worry about most during your quarantine period?
Please identify up to three elements that you consider most important.

- o Loneliness, lack of social contact, conflicts
- o Not being able to care for others
- o Losing my daily routine
- o Negative consequences of not exercising
- o Alcohol/drugs
- o Not being able to go outside
- o News about the pandemic
- o Poor support from health authorities
- o Lack of medical care (e.g., not getting help in the event of an emergency, lack of medication)
- o Lack of supplies (e.g., food)
- o Financial worries
- o Impact on work/study (e.g., being unable to cope with work, negative consequences at work/at school, job loss)
- o Symptoms/long-term effects/death or severe course of the illness in myself
- o Symptoms/long-term effects/death or severe course of the illness in my family members/friends/contact persons
- o Living situation (e.g., not enough space in the flat or house, no place to retreat to)
- o Having infected others
- o Testing positive for COVID-19/contracting COVID-19
- o Extension of quarantine
- o Accusations from others regarding my behaviour
- o Feelings of guilt regarding my behaviour
- o Other: ____
- o None

72) Did your quarantine period result in financial losses, or did you have higher financial expenses than usual?
You can select multiple answers.

- o No
- o Yes, due to my job (e.g., self-employed, hourly wage earner, reduced wages/commission/tips, short-time working, unpaid leave)
- o Yes, due to the loss of my job (e.g., laid off, no work/assignments, lack of pay)
- o Yes, due to living costs (e.g., delivery services, increased food costs)
- o Yes, due to higher utility bills (e.g., electricity consumption, household costs, water usage)

- Yes, due to special purchases (e.g., setting up a home office, medication, disinfectants, masks, online shopping)
- Yes, other (e.g., cancelled holiday, external pet care, activities for the child)

73) Did you make use of professional or voluntary support services?
You can select multiple answers.

- Yes, everyday support (neighbours, family, friends)
- Yes, delivery service
- Yes, City of Cologne helpline/COVID-19 hotline
- Yes, emergency medical service or GP by telephone
- Yes, hospital/clinic
- Yes, doctor (including emergency) or local ambulance service
- Yes, helpline
- Yes, pastoral care/self-help (groups)
- Yes, therapist or psychologist
- Yes, help for violence against women
- Yes, financial assistance
- Yes, support from employer
- Yes, other: _____________
- No, because I didn’t reach anyone.
- No, because I didn’t know of any.
- No

74) What further measures would you like to see, or would you have liked to see, following your quarantine period?
Please identify up to three elements that you consider most important.

- More social contact and the chance to talk to others in similar situations
- Stronger support from health authorities (e.g., earlier contact, friendlier staff, more regular phone calls, clearer information)
- Better medical care (e.g., support from my GP/hospital, post-COVID consultations, follow-up check-ups)
- Ensuring supplies via delivery services
- Financial support
- More support from work/university/school/daycare
- More testing (e.g., antibody and antigen tests after or during quarantine)
- Psychological support (e.g., helplines, support services)
- Support/care for children
- Different policies/behaviours from society (e.g., type of measures/lockdown, monitoring)
- Sports/leisure/entertainment options
- Less news about the pandemic
- More/easier access to information about the disease
- Other: __________________
- None

75) You indicated that you have made use of support services. Would you recommend them to others?

- Yes
- No

76) Why would you recommend the support services you have used?

- Because the staff were friendly
- Because it was helpful to speak to someone else
- Because I received useful information about quarantine
- Because it was beneficial for my mental well-being
- Because it provided me with financial support/relief/information
- Because it was helpful for my medical care
- Other: ___________________

77) Why would you not recommend the support services you have used?

- Because the staff were unfriendly
- Because it wasn't helpful
- Financial reasons
- Poor accessibility
- Other: ___________________

78) Were you excluded … due to your quarantine period?

a) … in your professional life …

- No
- Partially. How did this become apparent? _________________
- Yes. How did this become apparent? _________________

b) … in your private life …

- No
- Partially. How did this become apparent? _________________
- Yes. How did this become apparent? _________________

Eating behaviour during your quarantine period

79) Which of the following statements about your eating habits <u>during the quarantine period</u> apply to you?

	Yes	Partially	No
I split my daily food intake into three main meals and two snacks	O	O	O
I don't just eat at set mealtimes; I also eat in between if I feel hungry	O	O	O
Before every meal, I think carefully about exactly what and how much I want to eat	O	O	O
When choosing food, I follow the food pyramid or the food circle	O	O	O
I do something else while eating, such as reading or watching TV	O	O	O
I drink a glass of water or tea before a meal	O	O	O
I eat raw food before a meal (e.g., salad, fruit, vegetables)	O	O	O
Other: ________________________________	O	O	O

80) Did any of the following changes occur in your eating habits <u>during your quarantine period</u>?

Changes	Yes	No	Partially
Nothing	O	O	O
Eating healthier	O	O	O
Eating unhealthier	O	O	O
More snacks	O	O	O
Eating out of boredom	O	O	O
Eating for emotional support/out of frustration/for comfort	O	O	O
Eating less because I wasn't feeling well physically	O	O	O
Eating less due to COVID symptoms/loss of appetite and taste	O	O	O
Adapted my food choices to my quarantine situation (e.g., use-by dates, shopping lists, fewer impulse buys)	O	O	O
Other: ________________________________	O	O	O

81) Did any of the following changes occur regarding the food you eat <u>during your quarantine period</u>?

Nothing		O		
I eat more	O	O	O	***I eat less***
I eat more sweets	O	O	O	***I eat less sweets***
I eat more salty snacks	O	O	O	***I eat less salty snacks***
I eat more fruit	O	O	O	***I eat less fruit***
I eat more vegetables	O	O	O	***I eat less vegetables***
I eat more healthily	O	O	O	***I eat less healthily***
I eat earlier	O	O	O	***I eat later***
Food plays a bigger role in my life	O	O	O	***Food plays a lesser role in my life***
My eating habits/meals are more controlled/ regulated	O	O	O	***My eating habits/meals are less controlled/regulated***
I eat alone more often	O	O	O	***I eat with others more often***
I cook for myself more often	O	O	O	***I use delivery services more often***

82) During your quarantine period, did anything else change with regard to the food you eat?

- Yes: __________________
- No

83) Did your body weight change at all during your quarantine period?

- Yes
- No

84) How did your body weight change during your quarantine period?

- I lost ____________ kg.
- I gained ____________ kg.

About your lifestyle before the coronavirus pandemic

Exercise and relaxation

In this section, we would like to determine whether and how you were able to adapt your lifestyle in quarantine. Therefore, we will first ask about your usual activities before your quarantine period so that we have a point of comparison.

85) Were you generally physically active <u>before the pandemic</u>?

- o Yes
- o No

86) Were you physically active <u>in the 4 weeks leading up to your quarantine period</u>?

- o Yes
- o No

<u>Physical activity BEFORE your quarantine period</u>

87) What type of physical activity did you do <u>in the 4 weeks leading up to your quarantine period</u>?
You can select multiple answers.

- o Daily life activities (e.g., walking, housework, gardening)
- o Moderate physical activities (activities that *slightly* increase your breathing and heart rate and make you work up a *light* sweat)
- o Vigorous physical activities (activities that *significantly* increase your breathing and heart rate and make you sweat *considerably*)

Now think about your <u>daily life activities</u>.

88) On average, how many days per week (and for how long) did you engage in daily life activities <u>in the 4 weeks leading up to your quarantine period</u>?
(e.g., walking, housework, gardening, walking the dog)

Which activities? ___________________
How many days per week? ___________________
How many minutes per day? ____________________

Now think about your <u>moderate physical activities</u> (activities that *slightly* increase your breathing and heart rate and make you work up a *light* sweat).

89) On average, how many days per week (and for how long) did you engage in moderate physical activities <u>in the 4 weeks leading up to your quarantine period</u>?

Endurance activities:
(e.g., triathlon, jogging, cycling, swimming)

Which activities? ___________________
How many days per week? ___________________
How many minutes per day? ____________________

Strength training activities:
(e.g., fitness, EMS, calisthenics, circuit training)

Which activities? ___________________
How many days per week? ___________________
How many minutes per day? ____________________

Body and mind activities:
(e.g., yoga, Pilates, qigong)

Which activities? ___________________
How many days per week? ___________________
How many minutes per day? ____________________

Ball sports:
(e.g., football, basketball, volleyball)

Which activities? ___________________
How many days per week? ___________________
How many minutes per day? ____________________

Other sporting activities:
(e.g., dancing, Zumba, aerobics, hula hooping, rehabilitation/health-related exercise, stretching)

Which activities? ___________________
How many days per week? ___________________
How many minutes per day? ____________________

Now think about your <u>vigorous physical activities</u> (activities that *significantly* increase your breathing and heart rate and make you sweat *considerably*).

90) On average, how many days per week (and for how long) did you engage in vigorous physical activities <u>in the 4 weeks leading up to the quarantine period</u>?

Endurance activities:
(e.g., triathlon, jogging, cycling, swimming)

Which activities? ___________________
How many days per week? ___________________
How many minutes per day? ____________________

Strength training activities:
(e.g., fitness, EMS, calisthenics, circuit training)

Which activities? ___________________
How many days per week? ___________________
How many minutes per day? ____________________

Body and mind activities:
(e.g., yoga, Pilates, qigong)

Which activities? __________________
How many days per week? __________________
How many minutes per day? ___________________

Ball sports:
(e.g., football, basketball, volleyball)

Which activities? __________________
How many days per week? __________________
For how many minutes per day? ___________________

Other sporting activities:
(e.g., dancing, Zumba, aerobics, hula hooping, rehabilitation/health-related exercise, stretching)

Which activities? __________________
How many days per week? __________________
How many minutes per day? ___________________

91) Did your physical activity habits change <u>during your quarantine period</u>?

- o Yes
- o No

<u>Physical activity DURING your quarantine period</u>

92) Were you physically active <u>during your quarantine period</u>?

- o Yes
- o No

93) If not, why were you not physically active <u>during</u> your quarantine period?
You can select multiple answers.

- o Because I wasn't feeling well physically
- o Because I wasn't feeling well mentally
- o Because I don't have enough space
- o Because I didn't feel like it or was too lazy
- o Because I couldn't find any suitable options online
- o Because I don't have any sports equipment (e.g., dumbbells)
- o Because I had no idea what I could do
- o Because I generally don't engage in physical activities
- o Other: ___________________

94) **If yes, what type of physical activity did you do <u>during your quarantine period</u>?**
You can select multiple answers.

- Daily life activities (e.g., walking, housework, gardening)
- Moderate physical activities (activities that *slightly* increase your breathing and heart rate and make you work up a *light* sweat)
- Vigorous physical activities (activities that *significantly* increase your breathing and heart rate and make you sweat *considerably*)

Now think about your <u>daily life activities</u>.

95) **On average, how many days per week (and for how long) did you engage in daily life activities <u>during your quarantine period</u>?**
(e.g., walking, housework, gardening, walking the dog)

Which activities? ___________________
How many days per week? ___________________
How many minutes per day? ____________________

Now think about your <u>moderate physical activities</u> (activities that *slightly* increase your breathing and heart rate and make you work up a *light* sweat).

96) **On average, how many days per week (and for how long) did you engage in moderate physical activities <u>during your quarantine period</u>?**

Endurance activities:
(e.g., triathlon, jogging, cycling, swimming)

Which activities? ___________________
How many days per week? ___________________
How many minutes per day? ____________________

Strength training activities:
(e.g., fitness, EMS, calisthenics, circuit training)

Which activities? ___________________
How many days per week? ___________________
How many minutes per day? ____________________

Body and mind activities:
(e.g., yoga, Pilates, qigong)

Which activities? ___________________
How many days per week? ___________________
How many minutes per day? ____________________

Other sporting activities:
(e.g., dancing, Zumba, aerobics, hula hooping, rehabilitation/health-related exercise, stretching)

Which activities? __________________
How many days per week? __________________
How many minutes per day? ___________________

Now think about your <u>vigorous physical activities</u> (activities that *significantly* increase your breathing and heart rate and make you sweat *considerably*).

97) On average, how many days per week (and for how long) did you engage in vigorous physical activities <u>during your quarantine period</u>?

Endurance activities:
(e.g., triathlon, jogging, cycling, swimming)

Which activities? __________________
How many days per week? __________________
How many minutes per day? ___________________

Strength training activities:
(e.g., fitness, EMS, calisthenics, circuit training)

Which activities? __________________
How many days per week? __________________
How many minutes per day? ___________________

Body and mind activities:
(e.g., yoga, Pilates, qigong)

Which activities? __________________
How many days per week? __________________
How many minutes per day? ___________________

Other sporting activities:
(e.g., dancing, Zumba, aerobics, hula hooping, rehabilitation/health-related exercise, stretching)

Which activities? __________________
How many days per week? __________________
How many minutes per day? ___________________

<u>Physical activity OVER THE LAST 2 WEEKS</u>

98) Were you physically active <u>over the last 2 weeks</u>?

- ○ Yes
- ○ No

99) Was your physical activity behaviour over the last 2 weeks the same as your physical activity behaviour before your quarantine period?

- o Yes, my physical activity behaviour is now exactly the same as it was before my quarantine period
- o No, my physical activity behaviour is different than it was before my quarantine period

100) If not, why were you not physically active over the last 2 weeks?
You can select multiple answers.

- o Because I wasn't feeling well physically
- o Because I wasn't feeling well mentally
- o Because I don't have enough space
- o Because I didn't feel like it or was too lazy
- o Because I couldn't find any suitable options online
- o Because I don't have any sports equipment (e.g., dumbbells)
- o Because I had no idea what I could do
- o Because I generally don't engage in physical activities
- o Other: ___________________

101) What type of physical activity did you do <u>over the last 2 weeks</u>?
You can select multiple answers.

- o Daily life activities (e.g., walking, housework, gardening)
- o Moderate physical activities (activities that *slightly* increase your breathing and heart rate and make you work up a *light* sweat)
- o Vigorous physical activities (activities that *significantly* increase your breathing and heart rate and make you sweat *considerably*)

Now think about your <u>daily life activities</u>.

102) On average, how many days per week (and for how long) did you engage in daily life activities <u>over the last 2 weeks</u>?
(e.g., walking, housework, gardening, walking the dog)

Which activities? __________________
How many days per week? __________________
How many minutes per day? ___________________

Now think about your <u>moderate physical activities</u> (activities that *slightly* increase your breathing and heart rate and make you work up a *light* sweat).

103) On average, how many days per week (and for how long) did you engage in moderate physical activities <u>over the last 2 weeks</u>?

Endurance activities:
(e.g., triathlon, jogging, cycling, swimming)

Which activities? __________________
How many days per week? __________________
How many minutes per day? ___________________

Strength training activities:
(e.g., fitness, EMS, calisthenics, circuit training)

Which activities? __________________
How many days per week? __________________
How many minutes per day? ___________________

Body and mind activities:
(e.g., yoga, Pilates, qigong)

Which activities? __________________
How many days per week? __________________
How many minutes per day? ___________________

Ball sports:
(e.g., football, basketball, volleyball)

Which activities? __________________
How many days per week? __________________
How many minutes per day? ___________________

Other sporting activities:
(e.g., dancing, Zumba, aerobics, hula hooping, rehabilitation/health-related exercise, stretching)

Which activities? __________________
How many days per week? __________________
How many minutes per day? ___________________

Now think about your vigorous physical activities (activities that *significantly* increase your breathing and heart rate and make you sweat *considerably*).

104) On average, how many days per week (and for how long) did you engage in vigorous physical activities over the last 2 weeks?

Endurance activities:
(e.g., triathlon, jogging, cycling, swimming)

Which activities? __________________
How many days per week? __________________
How many minutes per day? ___________________

Strength training activities:
(e.g., fitness, EMS, calisthenics, circuit training)

Which activities? __________________
How many days per week? __________________
How many minutes per day? ___________________

Body and mind activities:
(e.g., yoga, Pilates, qigong)

Which activities? __________________
How many days per week? __________________
How many minutes per day? ___________________

Ball sports:
(e.g., football, basketball, volleyball)

Which activities? __________________
How many days per week? __________________
How many minutes per day? ___________________

Other sporting activities:
(e.g., dancing, Zumba, aerobics, hula hooping, rehabilitation/health-related exercise, stretching)

Which activities? __________________
How many days per week? __________________
How many minutes per day? ___________________

Relaxation activities BEFORE your quarantine period

105) Did you regularly practise relaxation activities in the 4 weeks leading up to your quarantine period?
(e.g., meditation, autogenic training, progressive muscle relaxation)

- o Yes
- o No

106) On average, how many days per week (and for how long) did you engage in relaxation activities in the 4 weeks leading up to your quarantine period?
(e.g., meditation, autogenic training, progressive muscle relaxation)

Which relaxation activities? __________________
How many days per week? __________________
How many minutes per day? ___________________

107) Did your relaxation activity habits change during your quarantine period?

- o Yes
- o No

Relaxation activities DURING your quarantine period

108) Did you regularly practise relaxation activities during your quarantine period?

- o Yes
- o No

109) If not, why did you not practise relaxation activities during your quarantine period?
You can select multiple answers.

- o Because I wasn't feeling well physically
- o Because I wasn't feeling well mentally
- o Because I don't have enough space
- o Because I didn't feel like it or was too lazy
- o Because I couldn't find any suitable options online
- o Because I don't have any equipment (e.g., yoga mat, meditation cushion, speaker)
- o Because I had no idea what I could do
- o Because I generally don't practise relaxation activities
- o Other: ___________________

110) If yes, on average, how many days per week (and for how long) did you engage in relaxation activities during your quarantine period?
(e.g., meditation, autogenic training, progressive muscle relaxation)

Which relaxation activities? __________________
How many days per week? __________________
How many minutes per day? ___________________

Relaxation activities OVER THE LAST 2 WEEKS

111) Did you regularly practise relaxation activities over the last 2 weeks?

- o Yes
- o No

112) Was your relaxation activity behaviour over the last 2 weeks the same as your relaxation activity behaviour before your quarantine period?

- o Yes, my relaxation activity behaviour is now exactly the same as it was before my quarantine period
- o No, my relaxation activity behaviour is different than it was before my quarantine period

113) If not, why did you not practise relaxation activities over the last 2 weeks?
You can select multiple answers.

- o Because I wasn't feeling well physically
- o Because I wasn't feeling well mentally
- o Because I don't have enough space
- o Because I didn't feel like it or was too lazy
- o Because I couldn't find any suitable options online
- o Because I don't have any equipment (e.g., yoga mat, meditation cushion, speaker)
- o Because I had no idea what I could do
- o Because I generally don't practise relaxation activities
- o Other: ___________________

114) If yes, on average, how many days per week (and for how long) did you engage in relaxation activities over the last 2 weeks?
(e.g., meditation, autogenic training, progressive muscle relaxation)

Which relaxation activities? __________________
How many days per week? __________________
How many minutes per day? ___________________

Sleeping habits

Sleep habits/disturbances BEFORE your quarantine period

115) How many hours of actual sleep did you get at night before your quarantine period?
This may be different than the number of hours you spent in bed.

Hours of sleep per night: _________

116) <u>Before your quarantine period</u>, how often did you suffer from… ?

	Never	Less than once a week	Once or twice a week	Three or more times a week
…trouble falling asleep	O	O	O	O
…trouble staying asleep	O	O	O	O

117) How would you rate your sleep quality overall <u>before your quarantine period</u>?

- o Very good
- o Fairly good
- o Fairly bad
- o Very bad

118) How often have you taken medicine to help you sleep (prescribed or "over the counter") <u>before your quarantine period</u>?

- o Never
- o Less than once a week
- o Once or twice a week
- o Three or more times a week

119) Did your sleeping habits/disturbances change <u>during your quarantine period</u>?

- o Yes
- o No

<u>Sleep habits/disturbances DURING your quarantine period</u>

Please answer the following questions with regard to your usual sleeping habits <u>during your quarantine period</u>.

120) How many hours of actual sleep did you get at night <u>during your quarantine period</u>?
This may be different than the number of hours you spent in bed.

Hours of sleep per night: _________

121) <u>During your quarantine period</u>, how often did you suffer from… ?

	Never	Less than once a week	Once or twice a week	Three or more times a week
…trouble falling asleep	O	O	O	O
…trouble staying asleep	O	O	O	O

122) How would you rate your sleep quality overall <u>during your quarantine period</u>?

- o Very good
- o Fairly good
- o Fairly bad
- o Very bad

123) How often have you taken medicine to help you sleep (prescribed or "over the counter") <u>during your quarantine period</u>?

- o Never
- o Less than once a week
- o Once or twice a week
- o Three or more times a week

124) Were your sleeping habits/disturbances <u>over the last 4 weeks</u> the same as your sleeping habits/disturbances before your quarantine period?

- o Yes
- o No

<u>Sleep habits/disturbances OVER THE LAST 4 WEEKS</u>

Please answer the following questions with regard to your usual sleeping habits <u>over the last 4 weeks</u>.

125) How many hours of actual sleep did you get at night <u>over the last 4 weeks</u>?
This may be different than the number of hours you spent in bed.

Hours of sleep per night: _________

126) <u>Over the last 4 weeks</u>, how often did you suffer from… ?

	**Never**	**Less than once a week**	**Once or twice a week**	**Three or more times a week**
…trouble falling asleep	O	O	O	O
…trouble staying asleep	O	O	O	O

127) How would you rate your sleep quality overall <u>over the last 4 weeks</u>?

- o Very good
- o Fairly good
- o Fairly bad
- o Very bad

128) How often have you taken medicine to help you sleep (prescribed or "over the counter") <u>over the last 4 weeks</u>?

- o Never
- o Less than once a week
- o Once or twice a week
- o Three or more times a week

129) On average, how many hours did you spend using screen-based media (e.g., computer, mobile phone, tablet, TV)… ?

a) professionally

…in the last 4 weeks leading up to your quarantine period	______ hours
…during your quarantine period	______ hours
…over the last 2 weeks	______ hours

b) personally

…in the last 4 weeks leading up to your quarantine period	______ hours
…during your quarantine period	______ hours
…over the last 2 weeks	______ hours

130) Did your screen time change during your quarantine period?

- o No
- o Yes, it increased
- o Yes, it decreased
- o Yes, other: ____________________

About your lifestyle during your quarantine period

131) How did you feel <u>during your quarantine period</u>?

	Completel y agree					Completel y disagree
I feel/felt restricted in terms of my physical ability.	O	O	O	O	O	O
I feel/felt exhausted.	O	O	O	O	O	O
I feel/felt tired.	O	O	O	O	O	O
I always feel/felt physically fit.	O	O	O	O	O	O

About your drinking behaviour during your quarantine period

132) How often do you usually drink alcohol <u>outside of your quarantine period</u>?

	Never	Once per month	2 or 3 times per month	2 or 3 times per week	4 or more times per week
Alcoholic drinks	O	O	O	O	O

133) Did this change <u>during your quarantine period</u>?

- Yes. How? ____________
- No

134) If you drink alcohol on a typical day, how many alcoholic drinks do you usually have <u>outside of your quarantine period</u>?
One alcoholic drink is equivalent to, for example, a small 0.33l bottle of beer, a small 0.125l glass of wine, a glass of sparkling wine, a double shot of liquor or a bottle of alcopop.

	1–2	3–4	5–6	7–9	10+
Alcoholic drinks	O	O	O	O	O

135) Did this change <u>during your quarantine period</u>?

- Yes. How? ____________
- No

136) How often did you drink more than 6 alcoholic drinks in a day <u>outside your quarantine period?</u>
One alcoholic drink is equivalent to, for example, a small 0.33l bottle of beer, a small 0.125l glass of wine, a glass of sparkling wine, a double shot of liquor or a bottle of alcopop.

	Never	Less than one per month	Once per month	Once per week	Daily or almost daily
More than 6 alcoholic drinks in a day	O	O	O	O	O

137) Did this change <u>during your quarantine period</u>?

- o Yes. How? ____________
- o No

Smoking

138) Do you smoke?

- o No
- o Yes

139) How long have you been smoking?

_________ years

140) How often do you smoke?

- o Daily
- o _______ days per week

141) How many cigarettes do you smoke? ____ per day

142) Did anything change in your smoking behaviour <u>during your quarantine period</u>?

- o Yes, I smoke(d) more
- o Yes, I smoke(d) less
- o Yes, other: ____
- o No

143) Did you contact health authorities yourself during your quarantine?
You can select multiple answers.

- o No
- o Yes, via the quarantine helpline
- o Yes, via the emergency number
- o Yes, via email
- o Yes, via the digital symptom diary
- o Yes (other): __________________

If you have any further comments or questions, please let us know here:
____
____
____

Thank you very much for your support!
